# Upregulation of the ESCRT pathway and multivesicular bodies accelerates degradation of proteins associated with neurodegeneration

**DOI:** 10.1080/27694127.2023.2166722

**Published:** 2023-01-22

**Authors:** Ron Benyair, Sai Srinivas Panapakkam Giridharan, Pilar Rivero-Ríos, Junya Hasegawa, Emily Bristow, Eeva-Liisa Eskelinen, Merav D Shmueli, Vered Fishbain-Yoskovitz, Yifat Merbl, Lisa M Sharkey, Henry L. Paulson, Phyllis I Hanson, Samarjit Patnaik, Ismael Al-Ramahi, Juan Botas, Juan Marugan, Lois S. Weisman

**Affiliations:** a Cell and Developmental Biology, University of Michigan, Ann Arbor, United States; Life Sciences Institute, University of Michigan, Ann Arbor, Michigan, United States; b Institute of Biomedicine, University of Turku, Turku, Finland; c Department of Systems Immunology, Weizmann Institute of Science, Rehovot, Israel; d Department of Neurology, University of Michigan, Ann Arbor, Michigan, United States; e Department of Biological Chemistry, University of Michigan School of Medicine, 1150 W. Medical Center Drive, Ann Arbor, Michigan, United States; f Department of Molecular and Human Genetics, Department of Molecular and Cellular Biology, Jan and Dan Duncan Neurological Research Institute, Houston, Texas, United States; g Division of Pre-Clinical Innovation, National Center for Advancing Translational Sciences, National Institutes of Health, 9800 Medical Center Drive, Rockville, Maryland 20850, United States

**Keywords:** ESCRT, Huntington, huntingtin, multivesicular bodies (MVB), NCT-504, Neurodegeneration, Tau

## Abstract

Many neurodegenerative diseases, including Huntington’s disease (HD) and Alzheimer’s disease (AD), occur due to an accumulation of aggregation-prone proteins, which results in neuronal death. Studies in animal and cell models show that reducing the levels of these proteins mitigates disease phenotypes. We previously reported a small molecule, NCT-504, which reduces cellular levels of mutant huntingtin (mHTT) in patient fibroblasts as well as mouse striatal and cortical neurons from an Hdh^Q111^ mutant mouse. Here, we show that NCT-504 has a broader potential, and in addition reduces levels of Tau, a protein associated with Alzheimer’s disease, as well as other tauopathies. We find that in untreated cells, Tau and mHTT are degraded via autophagy. Notably, treatment with NCT-504 diverts these proteins to multivesicular bodies (MVB) and the ESCRT pathway. Specifically, NCT-504 causes a proliferation of endolysosomal organelles including MVB, and an enhanced association of mHTT and Tau with endosomes and MVB. Importantly, depletion of proteins that act late in the ESCRT pathway blocked NCT-504 dependent degradation of Tau. Moreover, NCT-504-mediated degradation of Tau occurred in cells where Atg7 is depleted, which indicates that this pathway is independent of canonical autophagy. Together, these studies reveal that upregulation of traffic through an ESCRT-dependent MVB pathway may provide a therapeutic approach for neurodegenerative diseases.

**Abbreviations:** AD: Alzheimer’s disease CLEAR: Coordinated Lysosomal Expression and Regulation HTT: Huntingtin HD: Huntington’s disease MEF: Mouse embryonic fibroblasts HTT: Mutant Huntingtin MVB: Multivesicular bodies TFEB: Transcription factor EB

## Introduction

Huntington’s disease (HD) is an inherited, fatal neurodegenerative disease for which there is no effective treatment. HD is caused by an accumulation of the mutant huntingtin protein (mHTT), which contains an expanded polyglutamine stretch near its N-terminus. mHTT accumulation affects several cellular processes, such as protein synthesis, post-translational modification of proteins and transcriptional regulation. These changes ultimately result in cell death and loss of brain mass [1-6].

The accumulation of Tau has been linked to AD, as well as to other disease states, commonly known as tauopathies, where Tau proteins form neurofibrillary tangles, a hallmark of these pathological states [7,8]. Reduction in the levels of neurodegenerative-associated proteins such as mHTT and Tau mitigates disease phenotypes in animals. Thus, targeting Tau or mHTT for degradation has the potential to be an effective therapeutic intervention that could potentially work for multiple diseases [9-14].

The two main protein degradation pathways in mammalian cells are proteasomal and lysosomal degradation, with multiple routes leading to either pathway [15]. In proteasomal degradation, target proteins are modified with the addition of ubiquitin, a small protein capable of forming branched chains that are differentially identified by downstream effectors [16-18]. Ubiquitinated proteins can then be identified by the regulatory subunit of the proteasome complex, unfolded and proteolytically cleaved. Proteasomal degradation can also occur via 20S proteasome complexes independently of the 19S regulatory subunit and even independently of target protein ubiquitination [19,20]. This mode of proteasomal degradation has been implicated in the degradation of a number of neurodegeneration-associated proteins, including Tau and α-synuclein, which is linked to Parkinson’s disease pathology [21-23]. Proteasomal degradation has also been implicated in the degradation of mHTT and active proteasomes colocalize with mHTT inclusions and aggregates [24-27]. Tau and mHTT however, are not exclusively degraded by the ubiquitin-proteasome system and both undergo lysosomal degradation via macroautophagy (hereafter referred to as autophagy) [28,29]. Another pathway for protein degradation in lysosomes is via the ESCRT pathway on MVB. This pathway is critical in healthy cells for the removal of plasma membrane receptors that are ubiquitinated and targeted for degradation [30]. The ESCRT machinery includes the ESCRT-0 receptors and three ESCRT complexes (ESCRT-I/II/III), which coordinate to form intraluminal vesicles that include the selected cargoes. The Vps4 ATPase complex terminates the process of ESCRT-mediated substrate internalization and releases the ESCRT subunits to perform additional rounds of internalization. Thus, inhibition of Vps4, or ESCRT-III subunits inhibits MVB-mediated degradation of plasma membrane receptors [31-33].

While the proteasome, macroautophagy and the ESCRT pathway are each distinct, there is cross-talk between these pathways. For example, ESCRT subunits are necessary for autophagy [34-37]. Depletion of the ESCRT machinery leads to an accumulation of autophagosomes in cells, in a manner that may be species specific and remains largely unclear [38,39]. Recent studies have revealed that endosomal microautophagy, which also relies on some ESCRT subunits, provides another route to deliver proteins for lysosomal degradation [36,40,41]. Moreover, proteasomal function is required for some ESCRT-dependent pathways [42-44]. Thus, there is a high degree of interplay between the pathways that function in protein turnover.

We previously reported that the small molecule NCT-504, which was identified as a selective allosteric inhibitor of the phosphoinositide lipid kinase PIP4Kγ, reduced mHTT-exon1-polyQ in multiple cell types, and in addition reduced levels of mHTT in patient fibroblasts as well as in mouse striatal and cortical neurons. Moreover, NCT-504 resulted in an increase in autophagy. However, it was not clear whether the increase in autophagy accounted for the loss of mHTT [45].

Here we demonstrate that NCT-504 reduces the abundance of both mHTT and Tau. We show that this occurs via lysosomal degradation yet is independent of canonical autophagy. Instead, NCT-504 treatment results in the degradation of mHTT and Tau via ESCRT III and MVB. NCT-504 treatment increases the number of MVB. Furthermore, knockdown of the ESCRT-related proteins, VPS4A or CHMP4A, inhibit NCT-504 mediated degradation of mHTT and Tau. In addition, NCT-504 results in the translocation of transcription factor EB (TFEB) to the nucleus yet does not fully activate the canonical Coordinated Lysosomal Expression and Regulation (CLEAR) network. Surprisingly, NCT-504 reduces the cellular levels of TFEB, and intriguingly, shRNA mediated knockdown of TFEB is sufficient to increase MVB abundance and increase the degradation of Tau. Together, these results suggest that the degradation of some neurodegeneration-associated proteins can be increased via their diversion from autophagy to MVB, perhaps via endosomal microautophagy, and that the pathway(s) that utilize MVB should be further investigated as a potential approach to mitigate some neurodegenerative diseases.

## Results

### NCT-504 treatment reduces proteins associated with neurodegeneration

**Figure 1:**
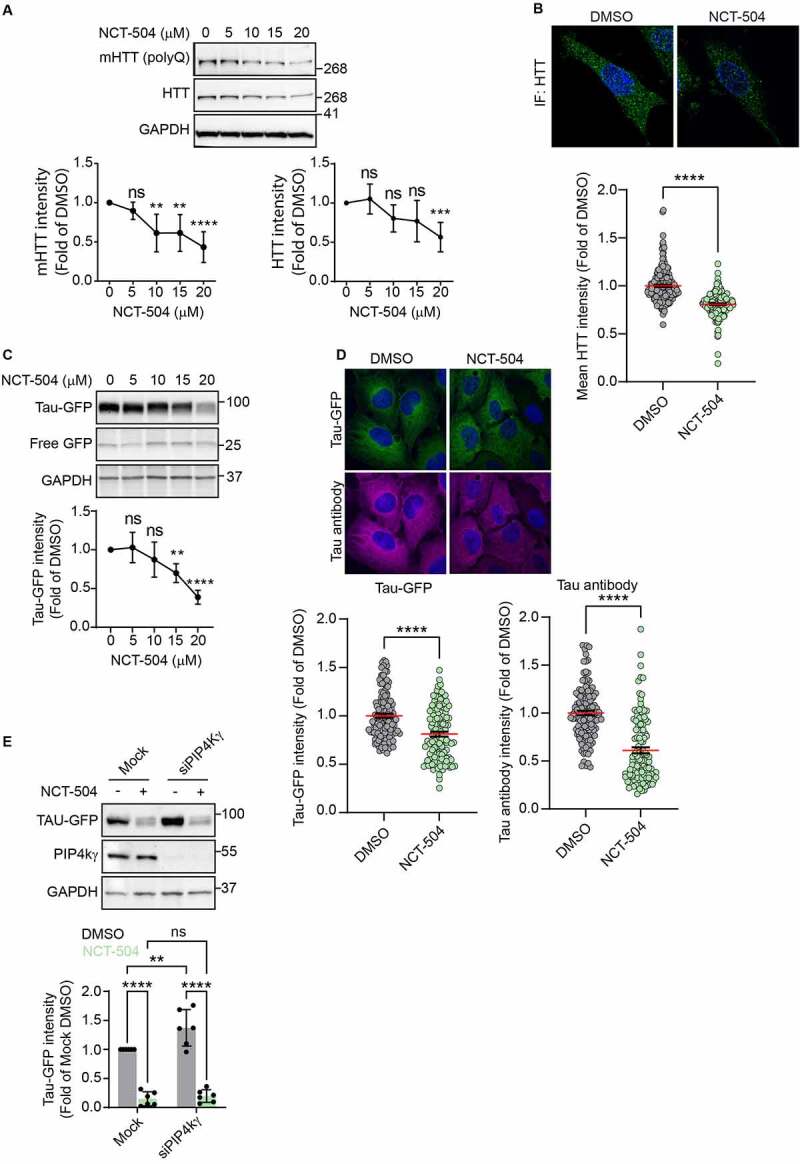
**– NCT-504 reduces the abundance of full length mHTT and Tau**. (**A**) Western blot and quantification of mHTT and HTT levels in primary MEF cells treated either with DMSO (0) or with NCT-504 at the indicated concentrations over 12 hours. N=7. (**B**) Immunofluorescence images and quantification of mean HTT intensity per cell in primary HD^Q200^ MEF cells, normalized to DMSO treated samples. N=3 with a total of 213 cells for DMSO treatment and 181 cells for NCT-504 treatment. (**C**) Western blot and quantification of Tau-GFP levels in DAOY Tau-GFP cells treated either with DMSO (0) or with NCT-504 at the indicated concentrations over 12 hours. N=6. For A and C, mean ± SD. One-way ANOVA used for statistical significance. (**D**) Immunofluorescence images and quantification of mean Tau-GFP or anti-Tau antibody staining intensities per cell, normalized to dsRed fluorescence and DMSO treated samples. 3 repeats with a total of 132 cells for DMSO treatment and 122 cells for NCT-504 treatment. For B and D, mean ± SEM. Unpaired, two-tailed t test used for statistical significance. (**E**) Western blot and quantification of Tau-GFP levels in DAOY cells, either mock-transfected or transfected with siRNA targeting PIP4Kγ and treated with either DMSO (-) or 20 µM NCT-504 (+) as indicated. N=6, mean ± SD. Two-way ANOVA used for statistical significance.

We previously showed that NCT-504 reduces the abundance and aggregation of an HTT-(exon 1) model protein as well as full-length mHTT in immortalized patient fibroblasts [45]. To further test the pathways whereby NCT-504 reduces mHTT, we generated primary fibroblasts expressing full-length mHTT isolated from a well-characterized knock-in mouse model, HD^Q200^, in which the CAG repeat in the HTT gene is expanded to approximately 200 repeats [46,47]. A time-course of treatment with 20 μM NCT-504, revealed that at 12 hours, there is a 46% decrease in mHTT, while the decreases at early time points were more modest (Figure S1A). Additionally, in a dose response experiment, 20 μM NCT-504 provided a 53% decrease in mHTT and also lowered wild-type HTT by 44% (Figure 1A). This fits with our earlier studies that NCT-504 impacts both HTT and mHTT [45]. Importantly, treatment of cells with 20 μM NCT-504 for 12 hours did not impact cell viability (Figure S1B).

In addition to western blot analysis, we developed an immunofluorescence (IF) assay for Huntingtin (HTT). We tested several huntingtin antibodies, and identified one, Abcam ab45169, which exhibited a strong signal in MEF infected with control shRNA, and had significantly less signal in MEF cells infected with shRNA targeting HTT (Figure S1C). Importantly, similar to western blot analysis of mHTT levels in HD^Q200^ fibroblasts treated with NCT-504, IF revealed a reduction in HTT levels during NCT-504 treatment (Figure 1B).

HTT levels could potentially decrease due to lower levels of HTT transcription. However, qPCR analysis revealed that NCT-504 did not significantly impact the transcript levels of HTT (Figure S1D). This suggests that the lowered levels of mHTT were likely due to a post-transcriptional pathway. In that scenario, other proteins associated with neurodegeneration may potentially be impacted by NCT-504 as well. Thus, we tested a second protein, Tau. Tau dysregulation is commonly observed in multiple types of neurodegenerative diseases, including AD, one of the most prevalent causes of neurodegeneration. To examine the effect of NCT-504 on Tau, we used human DAOY medulloblastoma cells, stably expressing dsRed-IRES-[WT]Tau:EGFP [48,49]. Importantly, as measured by western blot analysis, treatment with NCT-504 for 12 hours resulted in a dose-dependent reduction in Tau-GFP, where 20 μM NCT-504 resulted in a 61% decrease (Figure 1C). In addition, this reduction was observed by IF either probing for GFP or using an anti-Tau antibody (Figure 1D). That both Tau and mHTT were lowered by NCT-504 suggested that a general cellular mechanism was upregulated. Also, that Tau-GFP was lowered to a greater extent than mHTT, and that cell lines are easier to work with than primary MEF, led us to focus more on the Tau-GFP cells.

NCT-504 is an allosteric inhibitor of the phosphoinositide lipid kinase PIP4Kγ [45]. To determine whether depletion of PIP4Kγ has the same impact as treatment of cells with NCT-504, we depleted PIP4Kγ in Tau-GFP cells by siRNA transfection. Yet, these conditions did not cause a decrease in Tau-GFP. In fact, we observed a significant increase in Tau-GFP (Figure 1E). Moreover, even in the absence of PIP4Kγ, NCT-504 treatment reduced Tau-GFP levels (Figure 1E). In contrast, silencing of PIP4Kγ reduced mHTT levels in patient fibroblasts [45]. However, that NCT-504 mediated degradation can occur even in PIP4kγ-depleted medulloblastoma cells, suggested that additional targets are also impacted by this small molecule. Thus, we pursued studies to determine which pathway(s) are responsible for NCT-504-dependent reduction in mHTT and Tau.

Prior to testing protein degradation pathways, we first tested whether the intracellular levels of mHTT or Tau-GFP were lowered by secretion of these proteins. Cells have the potential to secrete multiple proteins associated with neurodegeneration. This is commonly observed for Tau, and has also been observed for mHTT [50]. Thus, we assessed the growth media of cells treated with either DMSO or NCT-504 by western blot analysis. Importantly, secretion of either mHTT or Tau-GFP was not detected in NCT-504 treated or control cells (Figure S1E, F). These findings turned our focus to determining whether upregulation of an intracellular degradation pathway is responsible for the reduction of these proteins.

### Both lysosomal and proteasomal activity are required for NCT-504 mediated degradation

Previous research into pathways that degrade mHTT and Tau under steady-state conditions indicated that multiple degradation pathways are involved in the clearance of these proteins. Specifically, both autophagy and proteasomal degradation have been associated with the clearance of mHTT, as well as Tau [51-53].

**Figure 2:**
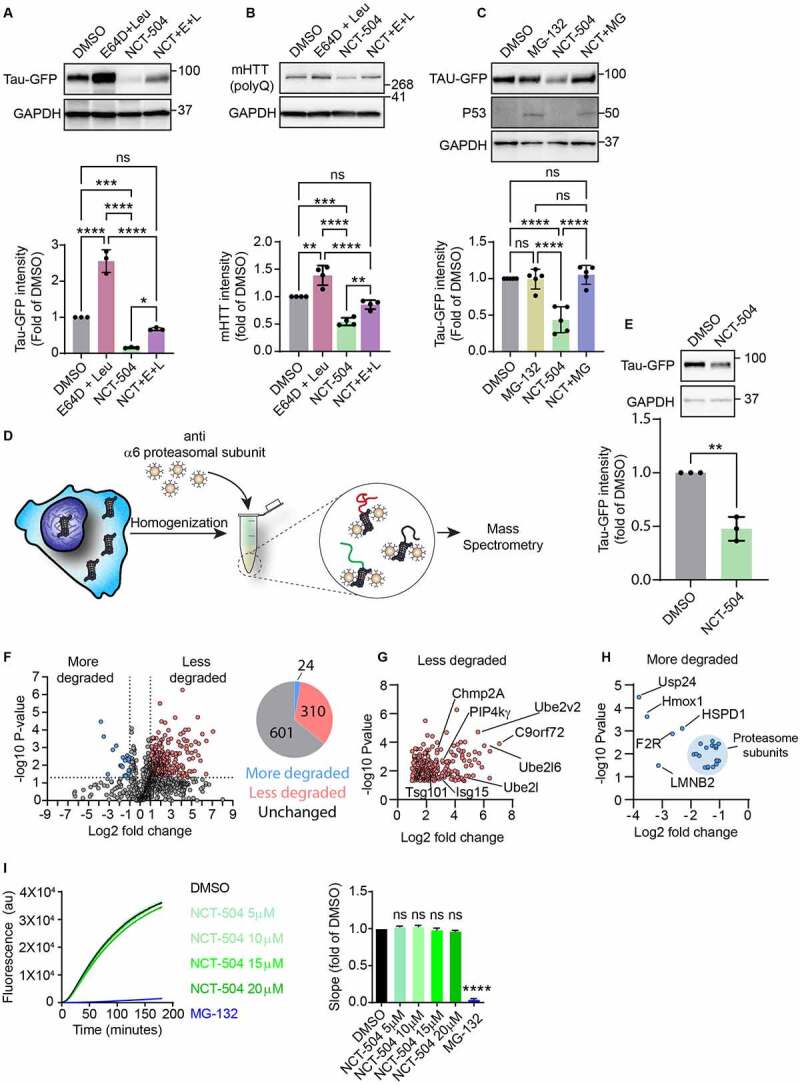
**- NCT-504 mediated degradation requires both lysosomal and proteasomal activity**. (**A**) Western blot and quantification of Tau-GFP levels in DAOY Tau-GFP cells treated either with DMSO or E64D (10 µg/ml) and leupeptin (100 µM) for 4 hours, with NCT-504 (20 µM) for 12 hours or with NCT-504 for 12 hours with E64D and leupeptin added for final 4 hours. N=3. (**B**) Same as A, carried out in primary HD^Q200^ MEF. N=4. (**C**) Western blot and quantification of Tau-GFP levels in DAOY Tau-GFP cells treated either with DMSO, with MG-132 (40 µM) for 4 hours, with NCT-504 (20 µM) for 12 hours or with NCT-504 for 12 hours with MG-132 added for final 4 hours. N=5. For A, B and C, mean ± SD. Two-way ANOVA used for statistical significance. (**D**) Schematic representation of the MAPP assay (**E**) Western blot and quantification of Tau-GFP levels in cells used for MAPP assay N=3, mean ± SD. Unpaired, two-tailed t test used for statistical significance. (**F**) Volcano plot representing MAPP assay results with proteins that became more abundant under NCT-504 treatment (blue), proteins that became less abundant (red) and proteins whose abundance did not change (grey), and pie-chart showing protein numbers. N=3 (**G**) Magnified view of proteins that were less abundant (less degraded) in MAPP under NCT-504 treatment. (**H**) Magnified view of proteins that were more abundant (more degraded) in MAPP under NCT-504 treatment. Proteasomal subunits circled in light-blue. (**I**) Quantification of suc-LLVY-AMC assay results over time and comparison of line slopes. N=3 with 3 technical repeats each, mean ± SEM. One-way ANOVA used for statistical analysis.

The degradation of autophagic cargoes requires lysosomal function. Thus, we tested whether inhibition of lysosomal proteases with a combination of E64D and leupeptin impaired NCT-504 mediated reduction of Tau-GFP or mHTT. Indeed, under basal conditions, without NCT-504, inhibition of lysosomal proteases caused an increase in the abundance of Tau-GFP (Figure 2A) and mHTT (Figure 2B). This indicates that lysosomal degradation contributes to the normal steady-state levels of Tau-GFP and mHTT. Importantly, in cells treated with lysosomal protease inhibitors for the final 4 hours of NCT-504 treatment, NCT-504-dependent reduction of Tau-GFP and mHTT was impaired. Similarly, addition of Bafilomycin A1, a vacuolar ATPase inhibitor, for the final 4 hours of treatment with NCT-504, impaired NCT-504-mediated degradation (Figure S2A). Together, these data suggest that NCT-504 mediated degradation requires active lysosomal proteases.

Proteasomal inhibition with MG132 (Figure 2C, S2C) or Bortezomib (Figure S2B) without NCT-504 treatment did not result in an increase in the abundance of Tau-GFP or mHTT. However, proteasomal inhibition blocked NCT-504 mediated degradation of Tau-GFP and mHTT. These findings suggest that under normal conditions, the steady-state levels of Tau and mHTT are predominantly independent of the proteasome. In contrast, NCT-504 mediated degradation requires both proteasomal and lysosomal activity.

Importantly, we found that NCT-504 does not stimulate proteasomal degradation of Tau and mHTT. We conducted a mass spectrometry analysis of proteolytic peptides (MAPP) assay to investigate the contents of proteasomes in control vs. NCT-504 treated cells [54,55] (Figure 2D, E, S2D). Briefly, cells were homogenized by freeze-thaw cycles and homogenates were cross-linked by DSP. Proteasomes were then harvested by immunoprecipitation using PSMA1 antibodies and their contents were eluted and analyzed by liquid chromatography – mass spectrometry. MAPP analysis detected a total of 935 proteins, eluted from proteasomes. Of these, NCT-504 treatment resulted in a decrease in 310 proteins (targeting to the proteasome was reduced), and an increase in 24 proteins (targeting to the proteasome was increased). The levels of the remaining 601 proteins did not change (Figure 2F). Peptides originating from Tau, or GFP in either control, or in NCT-504 treated proteasome samples were not detected (Table S1), despite these proteins being readily detected in mass-spectrometry of whole-cell samples (Table S2). In addition, only 31 transcription factors were detected, constituting ~3% of the detected proteins, while transcription factors constitute ~8% of the human genome [56]. Underrepresentation of transcription factors is consistent with the fact that these proteins are generally difficult to detect using mass spectrometry without dedicated methods [57]. Interestingly, PIP4Kγ abundance in proteasomes was decreased, indicating reduced degradation of this kinase (Figure 2G-H). This fits with the observation that in a screen of 450 kinases, NCT-504 bound selectively to PIP4Kγ [45]. As an orthogonal check of the MAPP assay results, we utilized suc-LLVY-AMC to test whether NCT-504 resulted in any changes in proteasome activity. However, NCT-504 at multiple concentrations did not inhibit proteasomal degradation (Figure 2I). That Tau-GFP and Tau were not detected in the proteasome assay, suggests that the role of the proteasome in NCT-504-dependent reduction of Tau-GFP may be related to an involvement of the proteasome in regulation of the pathway that degrades Tau.

### Atg7/Ulk1-dependent autophagy is not required for NCT-504 mediated degradation

**Figure 3:**
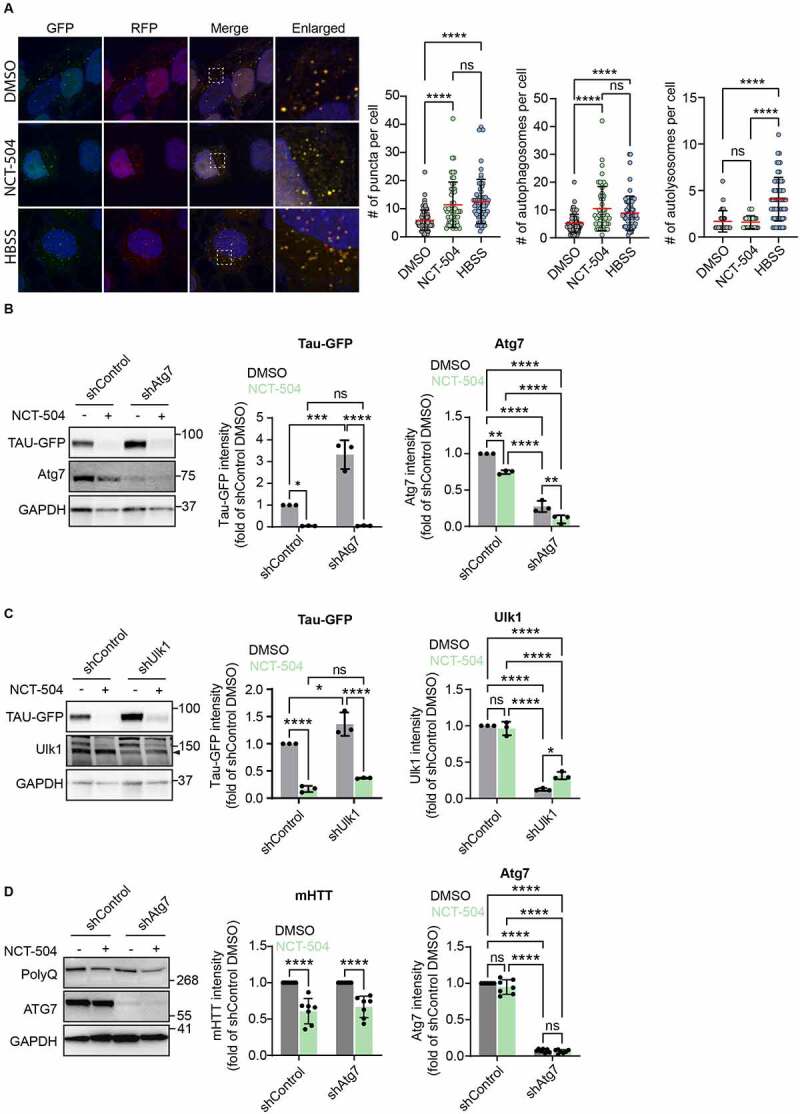
**- NCT-504 increases autophagic flux**. (**A**) Fluorescence and quantification of HeLa cells, stably expressing RFP-GFP-LC3 treated either with DMSO for 12 hours, NCT-504 (20 µM) for 12 hours or HBSS for 2 hours. 3 repeats with 92 cells for DMSO, 52 cells for NCT-504 and 63 cells for HBSS treatments. Mean ± SD. One-way ANOVA used for statistical significance. **Knockdown of proteins required for autophagy does not inhibit NCT-504 mediated degradation** (**B**) Western blot and quantification of Tau-GFP levels in DAOY cells, infected either with control shRNA (shControl) or with shRNA targeting Atg7 (shAtg7) and treated with either DMSO (-) or 20 µM NCT-504 (+) as indicated. N=3. (**C**) Western blot and quantification of Tau-GFP levels in DAOY cells, infected either with control shRNA (shControl) or with shRNA targeting Ulk1 (shUlk1) and treated with either DMSO (-) or 20 µM NCT-504 (+) as indicated. N=3. (**D**) Western blot and quantification of mHTT levels in primary MEF cells, infected either with control shRNA (shControl) or with shRNA targeting Atg7 (shAtg7) and treated with either DMSO (-) or 20 µM NCT-504 (+) as indicated. N=7. For A, B and C, mean ± SD. Two-way ANOVA used for statistical significance.

We previously reported that NCT-504 treatment resulted in an increase in autophagy [45]. To further test NCT-504-dependent effects on autophagy, we treated HeLa cells stably expressing RFP-GFP-LC3, with NCT-504 or HBSS as a starvation control. NCT-504 treatment resulted in an increase in total LC3 puncta as well as an elevation in the number of autophagosomes (Figure 3A). These observations could indicate either an increase in formation of autophagosomes, or a decrease in their clearance. Notably, NCT-504 treatment did not result in a similar, concomitant, increase in autolysosomes. These results raised the question of whether autophagy is the main pathway involved in NCT-504 mediated degradation.

To address this question, we used shRNA-mediated knockdown to target two major proteins required for autophagosome formation: Atg7 and Ulk1, which are each essential for canonical autophagy [58]. Note that Ulk1 is also involved in some forms of autophagy that are Atg7-independent [59]. Depletion of either Atg7 (Figure 3B) or Ulk1 (Figure 3C) increased the levels of Tau-GFP in cells, indicating that the normal degradation of Tau occurs through autophagy and requires these autophagy-related proteins. Importantly, NCT-504 mediated degradation of Tau-GFP or mHTT was not inhibited by depletion of Atg7 (Figure 3B, D) or Ulk1 (Figure 3C). These results indicate that while the steady state degradation of Tau occurs through autophagy, NCT-504 mediated degradation does not rely on autophagy but likely occurs via a different degradation mechanism.

### NCT-504 results in an increase in the number of MVB

**Figure 4:**
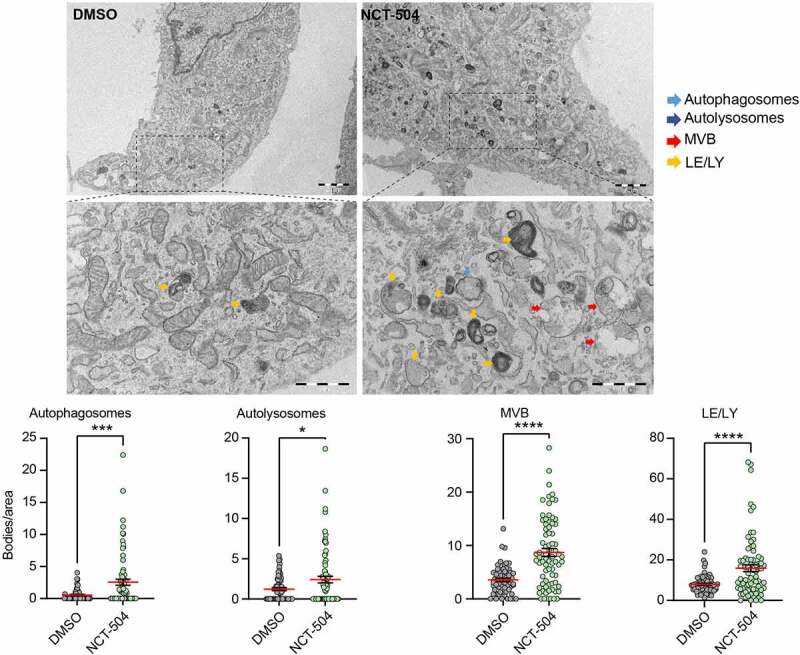
**– NCT-504 increases the abundance of endolysosomal organelles**. Representative transmission electron microscopy images of primary MEF cells treated either with DMSO (left) or 20 µM NCT-504 (right) and quantification of numbers of autophagosomes, autolysosomes, multivesicular bodies or late endosomes/lysosomes per cytoplasmic area. N=3 with 55 images quantified for DMSO and 77 for NCT-504 treatment. Unpaired, two-tailed t test used for statistical significance. Bar: 2 µm, Expanded bar: 1 µm.

To obtain a general view of potential changes in organelles of the endomembrane system during NCT-504 treatment, we treated primary wild-type as well as HD^Q200^ MEF cells, either with NCT-504 or DMSO, and analyzed them by transmission electron microscopy. Notably, in wild-type MEF, NCT-504 treatment for 12 hours resulted in a 4.8-fold increase in autophagosomes, a 1.9-fold increase in autolysosomes, a 2-fold increase in late endosomes/lysosomes and a 2.4-fold increase in MVB (Figure 4). There was a similar trend in HD^Q200^ MEF (Figure S3). While autophagy is considered the main route for degradation of proteins associated with neurodegeneration, it is possible that upregulation of an MVB-related pathway could lower some proteins. Plasma membrane receptors that are targeted via the MVB pathway to lysosomes, are first recognized on endosomes by the ESCRT machinery. The ESCRT machinery then invaginates the domain on endosomes where these cargoes are concentrated, and the resultant intraluminal vesicles provide a characteristic morphology that distinguish MVB from less mature endosomes. MVB fusion with lysosomes delivers the intraluminal vesicles and their contents for degradation [60]. Unlike canonical cargo, neither Tau nor mHTT are transmembrane proteins. However, mechanisms, such as endosomal microautophagy, which also requires some ESCRT subunits, may deliver these proteins to MVB [41,61]. Thus, the increase in MVB led us to further test whether MVB play a role in NCT-504 dependent degradation of mHTT and Tau.

**Figure 5:**
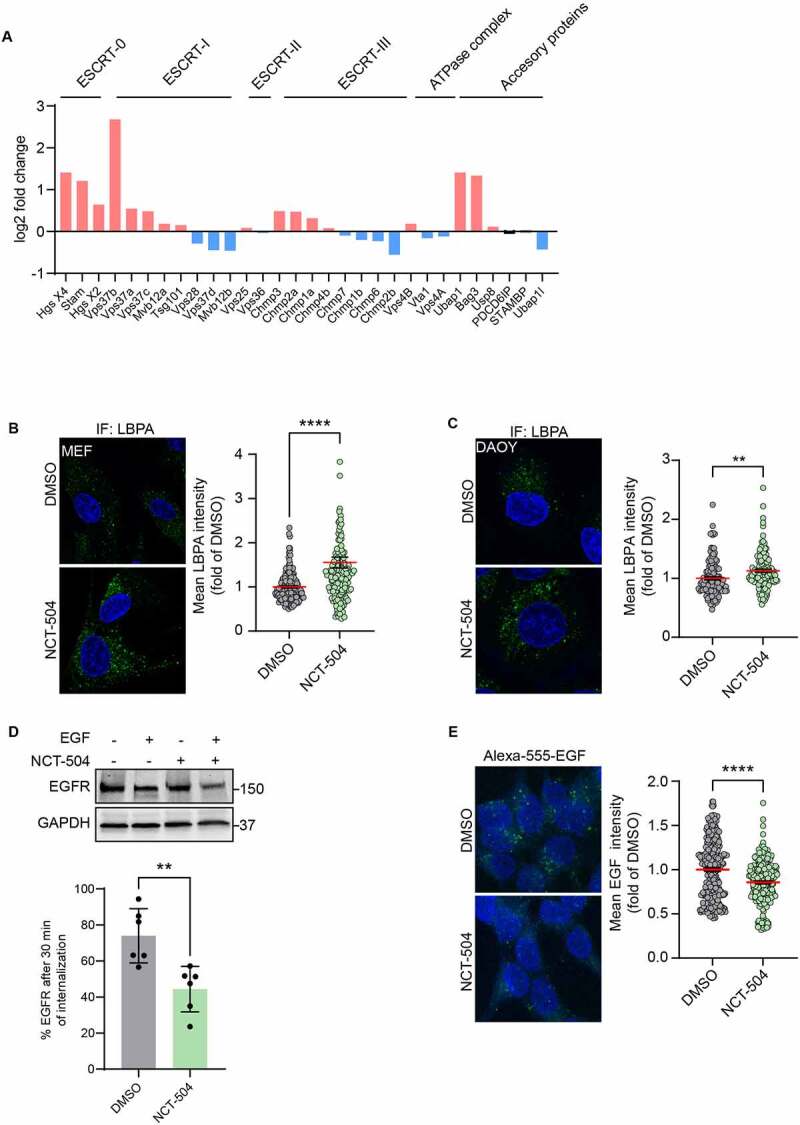
**– ESCRT transcripts and ESCRT-mediated degradation are upregulated by NCT-504**. (**A**) Transcriptional levels of ESCRT and associated genes obtained from RNAseq analysis. Upregulated genes are in red, downregulated genes in blue. (**B**) Immunofluorescence imaging and quantification of primary HD^Q200^ MEF cells treated either with DMSO or NCT-504 (20 µM) for 12 hours and immuno-stained for LBPA. N=3 repeats with 167 cells for DMSO and 161 cells for NCT-504 treatments. (**C**) Same as B, performed in DAOY Tau-GFP cells. N=3 repeats with 122 cells for DMSO and 129 cells for NCT-504 treatments. (**D**) Western blot and quantification of HEK 293 cells treated either with DMSO (-) or 20 µM NCT-504 (+) for 12 hours as indicated and exposed to EGF (+) or left unexposed (-) as indicated. N=6, mean ± SD. Unpaired, two-tailed t test used for statistical significance. (**E**) Immunofluorescence images of HEK 293 cells treated either with DMSO (-) or 20 µM NCT-504 (+) for 12 hours and exposed to Alexa 555-conjugated EGF. N=3 repeats with 341 cells for DMSO and 300 cells for NCT-504 treatment, mean ± SEM. Unpaired, two-tailed t test used for statistical significance.

RNAseq analysis of cells treated with either NCT-504 or DMSO for 12 hours (Table S3, Figure S4) showed that some ESCRT genes are transcriptionally upregulated by NCT-504 (Figure 5A). To assess changes in the number of MVB in a larger population of HD^Q200^ fibroblasts than what is feasible with transmission electron microscopy, we performed immunofluorescence to detect the MVB-specific phospholipid, LBPA (Figure 5B). Similarly, we used immunofluorescence, to detect MVB in DAOY Tau-GFP cells (Figure 5C). Notably, in both HD^Q200^ fibroblasts and DAOY cells, NCT-504 caused an increase in the abundance of MVB. To determine whether the increase in MVB abundance and ESCRT machinery transcription caused an increase in the degradation of a canonical MVB substrate, we tested if the internalization and degradation of EGFR, a well-established substrate, is affected by NCT-504. Indeed, in cells treated with NCT-504 and induced by addition of EGF, the cellular abundance of EGFR was reduced further than in cells treated with EGF and DMSO. This indicates that NCT-504 accelerates the internalization and degradation of EGFR (Figure 5D). In an orthogonal approach, we measured the internalization and degradation of Alexa-555 -conjugated EGF in cells either treated with NCT-504, or DMSO for 12 hours. Notably, NCT-504 treated cells had lower levels of Alexa-555-EGF, indicating an increase in degradation (Figure 5E). These results suggest that the upregulation in MVB and the ESCRT pathway impacts a canonical substrate of the ESCRT pathway.

### ESCRT-III is required for NCT-504 mediated degradation

Based on the elevated degradation of EGFR, we tested whether NCT 504-dependent degradation of Tau or mHTT occurs via the ESCRT pathway. The late steps of protein substrate internalization into MVBs occurs via the ESCRT-III machinery. Protein spirals formed by ESCRT-III, are required for the invagination of intralumenal vesicles [62,63]. The final sealing of the vesicles and the concomitant disruption and recycling of subunits of the ESCRT complex is catalyzed by the AAA-ATPase Vps4 [64,65]. Thus, knock-down of ESCRT-III subunits, or Vps4, have a similar result, and block transport through the ESCRT pathway.

**Figure 6:**
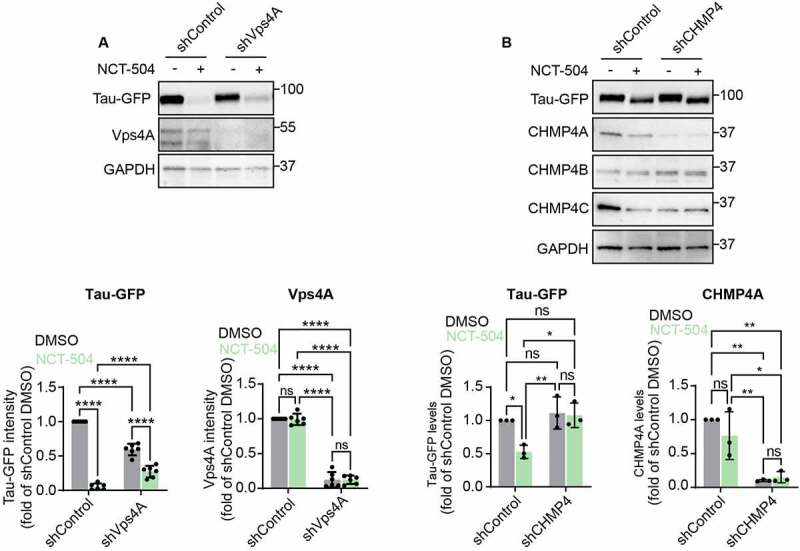
**– NCT-504 mediated degradation requires the ESCRT pathway**. (**A**) Western blot and quantification of Tau-GFP levels in DAOY cells, infected either with control shRNA (shControl) or with shRNA targeting Vps4A (shVps4A) and treated with either DMSO (-) or 20 µM NCT-504 (+) for 12 hours. N=6. Mean ± SD. (**B**) Western blot and quantification of Tau-GFP levels in DAOY cells, infected either with control shRNA (shControl) or with shRNA targeting CHMP4A, CHMP4B and CHMP4C (shCHMP4), treated with either DMSO (-) or 20 µM NCT-504 (+) for 12 hours. Note that only CHMP4A was effectively depleted. N=3, mean ± SD. For A and B, two-way ANOVA used for statistical significance.

Notably, while treatment with NCT-504 in control shRNA cells resulted in a depletion of Tau-GFP by 95%, knockdown of Vps4A impaired NCT-504-dependent effects; Tau-GFP was reduced by only 30% (Figure 6A). Note that knock-down of Vps4A on its own in the DMSO control, also resulted in a decrease in Tau-GFP.

We also tested the impact of knock-down of CHMP4 subunits, which are main ESCRT III subunits. While we used three lentiviruses, each of which targeted either CHMP4A, CHMP4B or CHMP4C, only CHMP4A was depleted. Importantly, depletion of CHMP4A blocked NCT-504 mediated degradation of Tau-GFP (Figure 6B). Importantly, in the absence of NCT-504, depletion of CHMP4A on its own did not impact Tau-GFP levels. Thus, NCT-504-dependent lowering of Tau requires both ESCRT III and Vps4A.

Similarly, in mHTT fibroblasts, NCT-504 lowered mHTT by 50%. In addition, knock-down of Vps4 on its own also lowered mHTT by approximately 50% when compared with the shControl virus alone. However, when compared with DMSO treatment of knock-down of Vps4A cells, NCT-504 treatment did not lower mHTT further (Figure S5A). Together these findings indicate that Vps4 has a role in NCT-504 dependent degradation of mHTT. In addition , these findings indicate that knock-down of Vps4 specifically, but not ESCRT III, in the absence of NCT-504, results the degradation of Tau-GFP and mHTT via an unknown mechanism.

We also tested the potential involvement of subunits from ESCRT-0 and ESCRT-I in NCT-504 mediated degradation. Knockdown of the ESCRT-0 subunits Hrs or STAM, or the ESCRT-I subunit Vps37B had no significant effect on the NCT-504 mediated degradation of Tau-GFP (Figure S6). These findings support the hypothesis that the ESCRT-III, but not ESCRT-0 or ESCRT-I, is important for NCT-504 mediated degradation of Tau-GFP.

**Figure 7:**
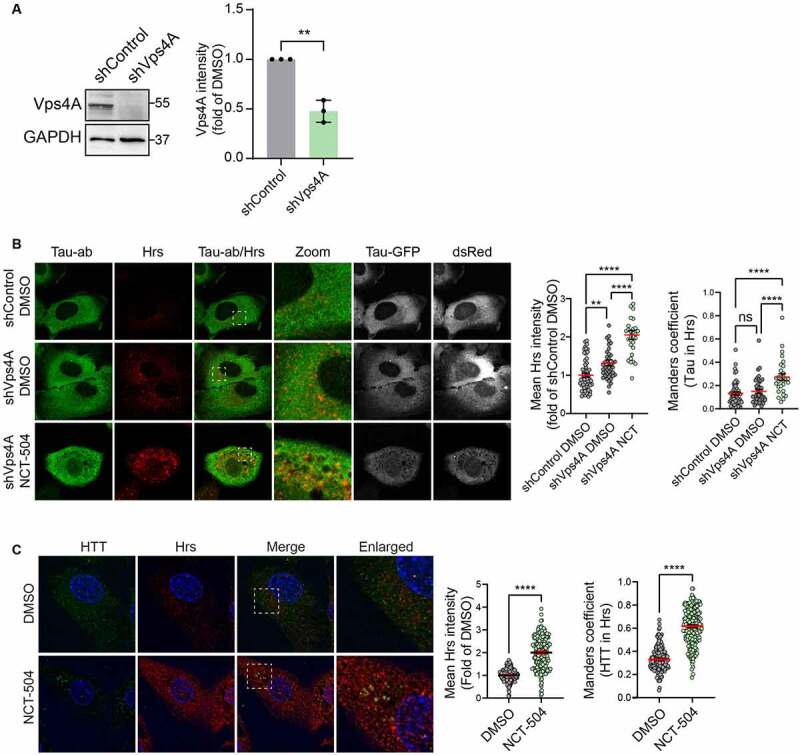
**– NCT-504 increases the colocalization of Tau/mHTT with MVB**. (**A**) Western blot and quantification of DAOY Tau-GFP cells infected with either control shRNA (shControl) or with shRNA targeting Vps4A (shVps4A). N=3, mean ± SD. Unpaired, two-tailed t test used for statistical significance. (**B**) Immunofluorescence imaging and quantification of Tau-GFP DAOY cells infected with either control shRNA (shControl) or with shRNA targeting Vps4A (shVps4A) and treated either with DMSO or 20 µM NCT-504 for 12 hours as indicated. N=3 repeats with 58 cells for shControl DMSO, 43 cells for shVps4A DMSO and 29 cells for shVps4A NCT-504. Mean ± SEM. One-way ANOVA used for statistical significance. (**C**) Immunofluorescence imaging and quantification of primary HD^Q200^ MEF cells treated either with DMSO or NCT-504 (20 µM) for 12 hours and immuno-stained for Hrs. N=3 repeats with 207 cells for DMSO and 202 cells for NCT-504 treatments. Mean ± SEM. Unpaired, two-tailed t test used to assess statistical significance.

Tau-GFP is predominantly cytosolic. To test whether NCT-504 treatment results in the targeting of Tau-GFP to MVB, we arrested MVB function by knocking down Vps4, and then used immunofluorescence to determine whether there was an increase in Tau-GFP colocalization with Hrs-containing MVB. Hrs, an ESCRT-0 subunit which is a receptor for some ESCRT cargoes, was used to label ESCRT-containing endosomes. NCT-504 treatment following Vps4A depletion increased Tau colocalization with Hrs (Figure 7A, B). Similarly, even without Vps4A knock-down, in HD^Q200^ MEF, NCT-504 caused an increase in the colocalization of HTT with Hrs (Figure 7C). NCT-504 also caused an increase in the colocalization of HTT with Vps35, a subunit of the retromer that resides on the same endosomes that participate in the ESCRT pathway [66] (Figure S5B). Note that in both the Tau-GFP cells, and HD^Q200^ MEF, there was a significant increase in the intensity of Hrs, and Vps35, which is consistent with the observed overall increase in endosomes and MVB (Figure 7B, C; Figure S5B). Thus, the increased colocalization of Tau and mHTT with MVB may be due at least in part to the total increase in MVB. Together, these results provide strong support for the hypothesis that NCT-504 diverts Tau-GFP from the autophagy pathway (Figure 3B-3D) to MVB and an ESCRT III-dependent pathway.

**Figure 8:**
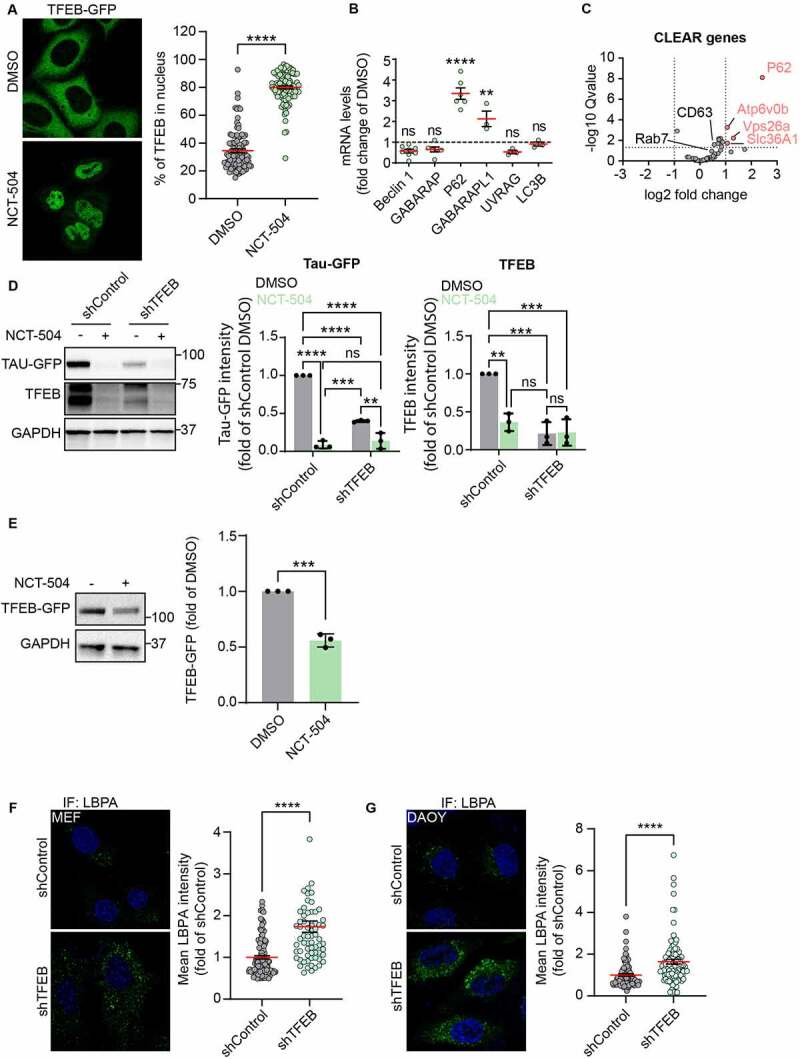
**– TFEB depletion increases MVB, accelerates Tau degradation**. (**A**) Fluorescence and quantification of HeLa cells, stably expressing TFEB-GFP treated either with DMSO for 12 hours or with NCT-504 (20 µM) for 12 hours. Quantification shows percentage of TFEB in the nucleus. 3 repeats with 104 cells in each condition. Mean ± SEM. Unpaired, two-tailed t test used for statistical significance. (**B**) qPCR analysis of TFEB-regulated genes in primary HD^Q200 MEF^ cells treated with 20 µM NCT-504 for 12 hours, compared to DMSO treated cells. N(Beclin1)= 8, N(GABARAP)= 6, N(P62)=6, N(GABARAPL1, UVRAG, LC3B)= 3. Mean ± SD. Unpaired, two-tailed t test used for statistical significance. (**C**) Volcano plot of TFEB target genes (CLEAR genes) quantified by RNAseq. Upregulated genes are in red, unchanged genes in grey. (**D**) Western blot and quantification of Tau-GFP levels in DAOY cells, infected either with control shRNA (shControl) or with shRNA targeting TFEB (shTFEB) and treated with either DMSO (-) or 20 µM NCT-504 (+) as indicated. N=3, mean ± SD. Two-way ANOVA used for statistical significance. (**E**) Western blot and quantification of TFEB-GFP cells in HeLa cells, stably expressing TFEB-GFP treated either with DMSO for 12 hours or with NCT-504 (20 µM) for 12 hours. N=3, mean ± SD. Unpaired, two-tailed t test used for statistical significance. (**F**) Immunofluorescence imaging and quantification of primary HD^Q200^ MEF cells infected either with control shRNA (shControl) or with shRNA targeting TFEB (shTFEB) and immuno-stained for LBPA. N=3 repeats with 98 cells for shControl and 99 cells for shTFEB samples. (**G**) Same as E, performed in DAOY Tau-GFP cells. N=3 repeats with 122 cells for shControl and 68 cells for shTFEB samples. For B-C, Mean ± SEM. Unpaired, two-tailed t test used for statistical significance.

We noted that NCT-504-mediated degradation did not occur until 12 hours after treatment, which suggests that NCT-504 effects could be due to transcriptional changes. Since NCT-504 caused an increase in the abundance of lysosomes and other endomembrane organelles (Figure 4), we tested whether NCT-504 activates TFEB, a major regulator of autophagy and lysosomal biogenesis. Upon activation, TFEB is dephosphorylated and is translocated from the cytosol to the nucleus [67], where TFEB activates the CLEAR gene network of lysosomal and autophagy-related genes [68,69]. We treated HeLa cells, stably expressing TFEB-GFP, with NCT-504 for 12 hours. Notably, under NCT-504 treatment, TFEB-GFP localized mainly in the nuclei (Figure 8A). Live-cell imaging further revealed that TFEB localization to the nucleus becomes apparent following 6 hours of treatment with NCT-504 (Figure S7). Notably, despite the nuclear translocation of TFEB, qPCR analysis showed that some, but not all, TFEB target genes were transcriptionally upregulated by NCT-504 treatment (Figure 8B). RNAseq analysis specifically of the CLEAR network further confirmed that only a subset of CLEAR genes were transcriptionally upregulated (Figure 8C, Table S4).

The observed changes in the numbers of lysosomes and MVB raised the possibility that TFEB activation could play a role in NCT-504 mediated degradation. To test this further, we knocked down TFEB. Surprisingly, even in the absence of NCT-504 treatment, knock-down of TFEB resulted in lowering of Tau-GFP by approximately 60% (Figure 8D). In addition, NCT-504 treatment of the TFEB depleted cells resulted in further reduction of Tau-GFP, by an additional 26%. Importantly, the further decrease of 26% was not as large as the reduction of 91% of Tau-GFP observed following NCT-504 treatment in cells that express TFEB. This raises the possibility that the NCT-504-dependent lowering of TFEB levels plays a role in NCT-504 dependent degradation of Tau-GFP. In support of this hypothesis, in two independent studies, we found that NCT-504 treatment substantially lowered Tau-GFP. NCT-504 treatment of shRNA control cells, lowered TFEB levels by 64%. Similarly, in HeLa cells stably expressing TFEB-GFP, NCT-504 treatment resulted in a 50% reduction in TFEB levels (Figure 8E). Moreover, TFEB depletion in either HD^Q200^ MEF or in Tau-GFP DAOY cells resulted in an increase in MVB (Figure 8F, G). Together, these data suggest that NCT-504 mediated degradation of mHTT and Tau-GFP is linked to a decrease in TFEB and a resultant increase in MVB.

Previous studies reported that overexpression of TFEB results in the degradation of Tau [70,71]. It is possible that NCT-504 treatment or increased TFEB expression, each lead to activated forms of TFEB that upregulate genes which accelerate Tau degradation.

## Discussion

The aberrant accumulation of aggregation-prone proteins is a hallmark of many neurodegenerative diseases. Reducing the abundance of these proteins is beneficial in both cell culture and in animal models [72-74]. One key method that is currently being pursued is anti-sense oligonucleotide silencing (ASO) of a specific target [75]. However, this approach needs further investigation. While FDA approval was provided for two ASO that target HTT, the patient trials had issues and were halted early. Many additional approaches are being pursued that target aggregation-prone proteins directly. For example, proteolysis-targeting chimera (PROTAC), links unwanted proteins with ubiquitin E3 ligases, to target them for proteasomal degradation [11,76-78]. However, both ASO and PROTAC therapies need to be tailored to an individual protein.

Alternative approaches that involve upregulation of protein degradation pathways via small molecules have the potential to be useful for a diverse range of proteins. The most advanced example is the acceleration of autophagy via inhibition of mTOR [79]. However, despite the effectiveness of mTOR inhibition for reducing the abundance of neurodegeneration-associated proteins, mTOR inhibition is highly toxic in human patients [80]. Thus, there is a major push for new approaches to effectively and safely reduce the abundance of neurodegeneration-associated proteins, either via alternative routes to upregulate autophagy, or via uncovering novel pathways to reduce toxic proteins.

**Figure 9:**
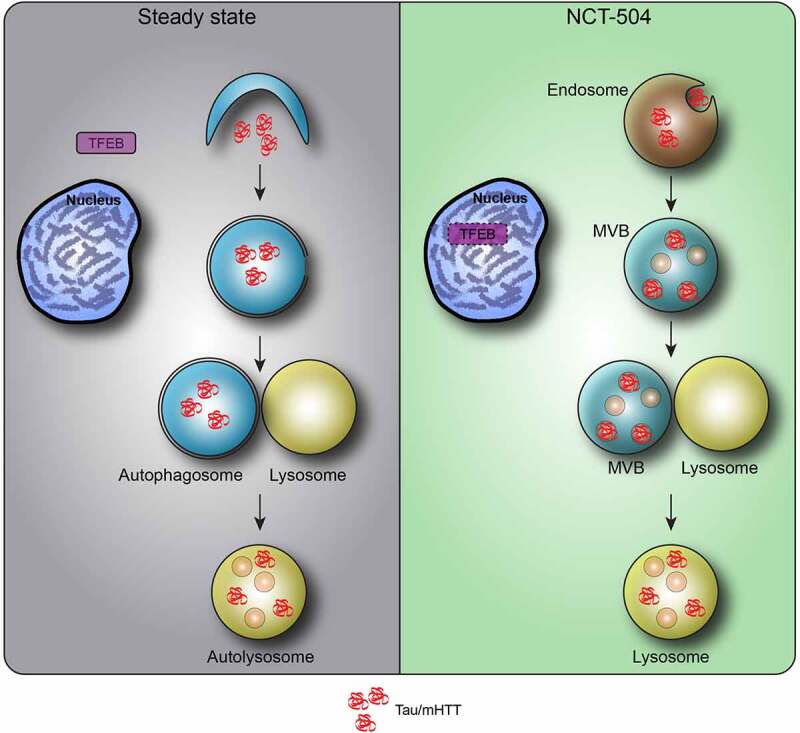
**– Model of NCT-504 action**. Under steady state, nutrient rich conditions, Tau and mHTT are turned over mainly by autophagy, and TFEB is found in the cytosol. Following NCT-504 treatment, Tau and mHTT are diverted from autophagy and directed to the ESCRT pathway on endosomes and MVB. Intriguingly, NCT-504 treatment results in TFEB translocation to the nucleus, as well as lowered levels of TFEB, and an increase is MVB and lysosomes. A direct causal link between NCT-504 dependent degradation, and NCT-504 dependent TFEB translocation, remains to be tested.

Here, we report that the small molecule, NCT-504 shifts the degradation of mHTT and Tau, from canonical autophagy to an endo-lysosomal pathway that requires ESCRT III (Figure 9). We find that NCT-504 treatment results in a proliferation of MVB. Importantly NCT-504 degradation of Tau-GFP and mHTT is impaired following knock-down of CHMP4A, a major subunit of ESCRT III, or Vps4A, the ATPase which is required to seal ESCRT-dependent intraluminal vesicles and recycle ESCRT subunits [62]. Note that NCT-504 is a tool compound and alters the transcription of a large number of targets (Table S3). Future studies using more specific compounds will be necessary to assess potential toxicity specifically associated with upregulation of MVB.

Interestingly, our findings reveal that proteasomal inhibition also inhibits NCT-504 mediated degradation, despite Tau peptides not being detected in the proteasomes. These findings fit with previous studies which show that proteasomal function is required for some ESCRT-dependent pathways [42-44,81,82], This is a likely explanation for the role of proteasomes. However, we also found that shRNA depletion of TFEB on its own resulted in the degradation of Tau-GFP (Figure 8D). Thus, alternatively, or in addition, the proteasome may be required for NCT-504 action because proteasomal degradation of TFEB may be a requisite step for NCT-504 dependent lowering of Tau-GFP and mHTT.

Notably, a few studies have revealed that endosomal microautophagy, also provides a route for lysosomal degradation [83]. For example, during starvation, selective autophagy receptors are degraded via endosomal microautophagy [41]. Importantly, there is some evidence that at low concentrations, exon 1 of mHTT may be degraded by a pathway that is consistent with endosomal microautophagy [84]. In addition, endosomal microautophagy can participate in the degradation of both wild-type and mutant Tau, however, chaperone-mediated autophagy (CMA) and macroautophagy, are the most common route [85]. While cytoplasmic Hsp8A participates in some endosomal microautophagy pathways [86], it is not required in others [41]. At this point the only proteins that are in common with all cases, are ESCRT III subunits and Vps4.

Additional evidence for Tau association with MVB comes from studies which utilized proximity ligation, and immunoprecipitation of overexpressed proteins to show that Tau interacts with Hrs on MVB. Importantly, knock-down of some ESCRT subunits or Rab35, a positive regulator of Hrs, was shown to impair Tau degradation [87]. In an independent study, overexpression of chaperone regulator 3 (Bag3), a positive regulator of Rab35, was shown to induce the colocalization of Tau with the ESCRT-III subunit CHMP2B, and accelerate Tau degradation [88]. Interestingly, Bag3 transcripts are upregulated by ~2.5-fold during NCT-504 treatment (Figure 5A). Whether Bag3 upregulation is associated with NCT-504-dependent degradation of Tau or mHTT remains to be elucidated.

Together, these previous studies along with results reported here suggest that multiple neurodegeneration-associated proteins have the potential to be degraded via an MVB to lysosome pathway, which perhaps is endosomal microautophagy. Importantly this pathway is accelerated by NCT-504 treatment. Notably, our studies indicate that this pathway can be upregulated using drug-like molecules.

Further research will be required to determine if NCT-504 treatment can target other aggregation-prone proteins to MVB, and whether TFEB inhibition can be used as a more proximal target to divert proteins linked to neurodegeneration to the MVB pathway. In addition, it will be important to uncover other upstream regulators that target proteins to MVB for lysosomal degradation.

## Materials and methods

### Cell lines

Primary mouse embryonic fibroblasts were prepared from either WT C57BL/6J or mHTT knock-in mice [89]. P0 mouse pups were sacrificed and segmented, limbs were then washed in RPMI media. Limbs were then incubated overnight at 37°C in RMPI/ 15% FBS/ penicillin/ streptomycin and collagenase type II (2,000 U). Samples were then centrifuged for 2 minutes at 600 X g. Medium was removed and samples were washed with RPMI media. Samples were then resuspended in RMPI/ 15% FBS/ penicillin/ streptomycin, plated in 10 cm cell culture dishes and incubated at 37°C with 5% CO_2_. Once cells reached ~90% confluence, they were split once into multiple 10 cm cell culture plates which were incubated at 37°C with 5% CO_2_ until they reached ~90% confluence. Cells were then trypsinized, aliquoted and frozen in DMEM/ 15% FBS/ penicillin/ streptomycin and 10% DMSO. Once thawed, all MEF were grown in DMEM/ 15% FBS/ penicillin/ streptomycin. To validate CAG repeat length, DNA was extracted from tail sections of mice (DNAeasy Blood and Tissue Kit, Qiagene 69504) and sequenced by Laragen Inc. (Culver City, CA USA). All MEF used had between 196 and 203 CAG repeats.

The DAOY medulloblastoma cell line stably expressing DsRed-IRES-Tau:EGFP (wild-type Tau-411, the 2N4R isoform) were previously described and characterized [48,49]. This cell line was grown in DMEM/ 10% FBS/ penicillin/ streptomycin at 37°C with 5% CO_2_. HEK293-T cells were grown in DMEM/ 10% FBS/ penicillin/ streptomycin at 37°C with 5% CO_2_.

### Animal use

All MEF cell lines used in this study were produced in the Weisman laboratory at the University of Michigan from male and female C57BL/6J HdhQ200 knock-in mice [89]. Mice were housed in cages, separated by sex and provided food and water *ad libitum*. All mice were held in specific pathogen free conditions with 12-hour light/dark cycles. All procedures were conducted in compliance with the guide for care and use of laboratory animals.

### Reagents

NCT-504 was solubilized in DMSO (Tocris, 3176) [45]. Unless stated otherwise, cells were treated with 20 µM NCT-504 for 12 hours; E64D (Sigma, E8640) was used at 10 µg/ml for 4 hours. Leupeptin (Sigma, L2884) was used at 100 µM for 4 hours; MG-132 (Enzo life sciences international, BML-PL102-0025) was used at 40 µM for 4 hours; HBSS (Invitrogen, 14175095) was used for starvation for 2 hours; Bafilomycin A1 (Cayman chemicals, 11038) was used at 100nM for 4 hours; Bortezomib (Sigma, 5043140001) was used at 100nM for 12 hours; EGF-555 (Invitrogen, E35350); Collagenase type II (Worthington, LS004202), TrypLE express (Gibco, 12605-010) DMEM (Invitrogen, 11995065), Fetal calf serum (Sigma, F4135).

### Antibodies

PolyQ (Millipore, MAB1574); HTT (Millipore, MAB2166); GAPDH (Thermo, AM4300); GFP (Abcam, ab32146); (HTT (Abcam, ab45169); Tau (Abcam, ab76128); PIK4K2C (Proteintech, 17077-1ap); Tp53 (Cell signaling, 2524); PSMA1 (Hybridoma kindly provided by K.G. Tanaka, University of Tokyo); mTOR P-S2448 (Cell signaling, 5536); mTOR (Cell signaling, 2983); S6K P-T389 (Cell signaling, 9205), S6K (Cell signaling, 2708); TFEB (Cell signaling, 4240); Akt P-S473 (Cell signaling, 4051); Akt P-T308 (Cell signaling, 13038); Akt (Cell signaling, 9272); Atg7 (Cell signaling, 8558); Ulk1 (Cell signaling, 8054), LBPA (Echelon, Z-PLBPA); STAM (Proteintech, 12434-1-AP); Hrs (Proteintech, 10390-1-AP); Vps37B (Proteintech, 15653-1-AP); Tsg101 (Santa Cruz, Sc-7964); EGFR (Cell signaling, 2232); Vps4A (Santa Cruz, sc-393428); CHMP4A (Kindly provided by Phyllis Hanson, University of Michigan); CHMP4B (Kindly provided by Phyllis Hanson, University of Michigan); CHMP4C (Origene, TA890004S); Donkey anti mouse hrp (Jackson immuno-research, 715-035-150); Goat anti rabbit hrp (Jackson immuno-research, 111-035-144); Goat anti mouse 488 (Thermo, A32723); Goat anti rabbit 594 (Thermo, A-11012); Goat anti mouse 647 (Thermo, A32728).

### Lentivirus production, siRNA and shRNA

All shRNA lentiviral particles were produced in HEK 293T cells, transfected with packaging vectors pMD2.G and psPAX2 along with relevant shRNA in pLKO.1 vector using Fugene 6 transfection reagent (Promega, E2691). Viral particles were harvested over two days in DMEM/ 40% FBS, centrifuged for 5 minutes at 1,000 X g to clear debris and then aliquoted for freezing at -80°C. For infections, cells were plated in 10 cm cell culture dishes and allowed to grow to a density of ~25% in DMEM/ 10% FBS/ penicillin/ streptomycin. Cells were then re-fed with fresh medium supplemented with 7 µg/ml polybrene (Sigma, TR-1003-G) and infected with 2 ml of viral preparation and allowed to incubate at 37°C with 5% CO_2_ over 24 hours. The cells were then exposed to selection medium containing 3 µg/ml puromycin (Sigma, P8833) for an additional 48 hours and then passaged for the experiments. siRNA transfections were done using Fugene 6 following manufacturer’s instructions, using 5nM of siRNA.

**Table:** 

Type	Species	Target	Company	Cat#	Sequence
siRNA	Human	PIP4K2C	Dharmacon	L-004535-00	Smartpool
shRNA	Human	TFEB	Sigma	TRCN0000440038	CCGGTGGCAACAGTGCTCCCAATAGCTCGAGCTATTGGGAGCACTGTTGCCATTTTTTG
shRNA	Human	Atg7	Sigma	TRCN0000007584	CCGGGCCTGCTGAGGAGCTCTCCATCTCGAGATGGAGAGCTCCTCAGCAGGCTTTTT
shRNA	Mouse	Atg7	Sigma	TRCN0000375444	GCCAACATCCCTGGATACAAG
shRNA	Human	Ulk1	Sigma	TRCN0000000835	CCGGGCCCTTTGCGTTATATTGTATCTCGAGATACAATATAACGCAAAGGGCTTTTT
shRNA	Human	Vps4A	Sigma	TRCN0000159521	CCGGGCCTGCTGAGGAGCTCTCCATCTCGAGATGGAGAGCTCCTCAGCAGGCTTTTT
shRNA	Human	CHMP4A	Sigma	TRCN0000319265	GACAGAGAAGATACTGATCAA
shRNA	Human	CHMP4B	Sigma	TRCN0000180330	CGGCACATTATCAACCATCGA
shRNA	Human	CHMP4C	Sigma	TRCN0000147799	GCGATGAAATCTGTTCATGAA
shRNA	Mammalian	Non-targeting	Sigma	SHC002	

### Western blotting and quantification

SDS-PAGE was carried out using Criterion™ TGX™ precast 4-15% gradient 18-well midi gels (Bio-Rad, 5671084). Samples were prepared in 4X Laemmli sample buffer (Bio-Rad, 1610747) and 10% β-mercapto ethanol (Acros organics, 149582500). Gels were run in Criterion™ cell (Bio-Rad, 1656001) using PowerPac™ power supply (Bio-Rad, 1645050), SDS running buffer (Tris base: 0.2501 M, Glycine: 1.924 M, SDS: 0.03467 M) for 70 minutes at 150 constant volts. Transfer to membranes was carried out in a Criterion™ Blotter (Bio-Rad, 1704071), transfer buffer (Tris base: 25 mM, Glycine: 192 mM, Methanol: 20% v/v) over 1 hour at 100 constant volts for samples of DAOY Tau-GFP, or 3 hours at 350 constant mA for mHTT MEF. All transfers were carried out at 4°C onto 0.45 µM nitrocellulose membranes (Bio-Rad, 1620167). Membranes were incubated with antibodies overnight at 4°C, washed three times and incubated with secondary Hrp-conjugated antibodies at room temperature for 1 hour. Membranes were then exposed to ECL (Amersham Cytiva, RPN2106) or ECL prime (Amersham Cytiva, RPN2236) prior to imaging using a ChemiDoc™ touch gel imaging system (Bio-Rad, 1708370). Protein size ladders used were either precision plus protein dual color (Bio-Rad, 161-0374) or Himark pre-stained HMW protein standard (ThermoFisher, LC5699). All images were quantified using Image Lab software (Bio-Rad, version 6.0.1).

### Immunofluorescence, live cell imaging and image analysis

For immunofluorescence, cells were fixed in 4% paraformaldehyde (Fisher, BP531-500 at room temperature over 15 minutes and permeabilized in 0.5% Triton/PBS at room temperature for 15 minutes. Cells were then incubated with 2% BSA (Lampire, 7500806) for 10 minutes and incubated with primary antibodies, diluted in 2% BSA for 1 hour. Cells were then washed three times in PBS/ BSA (2%) before being incubated with secondary antibodies and Hoechst (Invitrogen, H3570) for 30 minutes. Images were obtained on an SP8 laser scanning confocal microscope with X63 oil objective (Leica microsystems, USA). Live cell microscopy was carried out using DeltaVision Restoration system (Applied Precision) using an inverted epifluorescence microscope (IX-71; Olympus) with a charge-coupled device camera (Cool-SNAP HQ; Photometrics). Image analysis were performed using CellProfiler software [90] (Broad institute, cellprofiler.org).

### Secretion assay

Cells were plated in 60 mm cell culture dishes (SPL, 20060) in serum-free DMEM and incubated for 12 hours at 37°C with 5% CO_2_ before being treated either with NCT-504 (20 µM) or DMSO for an additional 12 hours. At the end of incubation, growth media and corresponding cells were harvested separately. 20% of total media was precipitated by trichloroacetic acid (BDH, BDH7372-2). Media and cells were analyzed by SDS-PAGE.

### Cell death assay

The LIVE/DEAD™ viability/ cytotoxicity kit for mammalian cells as per protocol (ThermoFisher, L3224) was utilized to determine cell death.

### Mass-spectrometry analysis of proteolytic peptides (MAPP)

Cells, treated either with NCT-504 (20 µM) or DMSO for 12 hours were harvested, pelleted and frozen as dry pellets at -80°C. Cells were then shipped on dry ice to the Weizmann Institute of Science for the MAPP procedure, as previously published [54]. Briefly, cells were homogenized and homogenates were then cross-linked. Proteasomes were immunoprecipitated with PSMA1 antibodies and peptides were eluted, validated by SDS-PAGE and used for mass-spectrometry analysis.

### Purification of proteasome complexes

Cells were lysed with 25 mM HEPES, pH 7.4, 10% glycerol, 5 mM MgCl2, 1 mM ATP, and 1:400 protease-inhibitor mixture (Calbiochem), then homogenized through freeze–thaw cycles and passed through a needle. The lysates were cleared by 30-min centrifugation at 21,130g at 4 °C. Pellets were lysed again with 0.5 mM ammonium persulfate to enrich nuclear fraction followed be centrifugation. Mixed lysates were treated with 2 mM 1,10-phenanthroline (Sigma), cross-linked with 0.5 mM DSP (Thermo Fisher Scientific) for 30 min at room temperature, and quenched in 100 mM Tris-HCl, pH 8, 5 mM L-cysteine for 10 min at room temperature. For immunoprecipitation, the lysates were then incubated with Protein G–Sepharose beads (Santa Cruz) with antibodies to PSMA1 and eluted with 100 mM Tris-HCl, pH 8, 8M urea and 50 mM DTT for 30 min at 37 °C. Subsequently, 1% trifluoroacetic acid (TFA) was added. Aliquots of each elution fraction were analyzed by SDS–PAGE to evaluate yield and purity.

### Purification and concentration of proteasome peptides

A critical step in our procedure is the separation of peptides from the proteins eluted in the proteasome pulldown. MAPP analyzes endogenously cleaved peptides, whereas the proteasome complex and associated proteins are physically excluded. Immunoprecipitated proteasomes and their encompassed peptides were loaded on C18 cartridges (Waters) that were prewashed with 80% acetonitrile (ACN) in 0.1% TFA, then washed with 0.1% TFA only. After loading, the cartridges were washed with 0.1% TFA. Peptides were eluted with 30% ACN in 0.1% TFA. Protein fractions were eluted with 80% ACN in 0.1% TFA.

### Mass spectrometry sample processing and analysis

Protein fraction after proteasome purification; Proteins were denatured by 8M Urea for 30 min at room temperature, reduced with 5 mM dithiothreitol (Sigma) for 1hr at room temperature and alkylated with 10 mM iodoacetamide (Sigma) in the dark for 45 min at room temperature. Samples were diluted to 2M urea with 50mM ammonium bicarbonate. Proteins were then subjected to digestion with trypsin (Promega; Madison, WI, USA) overnight at 37°C at 50:1 protein:trypsin ratio, followed by a second trypsin digestion for 4 hr. The digestions were stopped by addition of trifluroacetic acid (1% final concentration). Following digestion, peptides were desalted using Oasis HLB, μElution format (Waters, Milford, MA, USA). The samples were vacuum dried and stored in -80°C until further analysis.

Total proteomics; Lysates in 5% SDS in 50 mM Tris-HCl were incubated at 96 °C for 5 min, followed by six cycles of 30s of sonication (Bioruptor Pico, Diagenode, USA). Proteins were reduced with 5 mM dithiothreitol and alkylated with 10 mM iodoacetamide in the dark. Each sample was loaded onto S-Trap microcolumns (Protifi, USA) according to the manufacturer’s instructions. In brief, after loading, samples were washed with 90:10% methanol/50 mM ammonium bicarbonate. Samples were then digested with trypsin for 1.5 h at 47 °C. The digested peptides were eluted using 50 mM ammonium bicarbonate; trypsin was added to this fraction and incubated overnight at 37 °C. Two more elutions were made using 0.2% formic acid and 0.2% formic acid in 50% acetonitrile. The three elutions were pooled together and vacuum-centrifuged to dry. Samples were kept at −80 °C until analysis.

### Liquid chromatography mass spectrometry

Peptide fraction; ULC/MS grade solvents were used for all chromatographic steps. Each sample was loaded using split-less nano-Ultra Performance Liquid Chromatography (10 kpsi nanoAcquity; Waters, Milford, MA, USA). The mobile phase was: A) H2O + 0.1% formic acid and B) acetonitrile + 0.1% formic acid. Desalting of the samples was performed online using a reversed-phase Symmetry C18 trapping column (180 µm internal diameter, 20 mm length, 5 µm particle size; Waters). The peptides were then separated using a T3 HSS nano-column (75 µm internal diameter, 250 mm length, 1.8 µm particle size; Waters) at 0.35 µL/min. Peptides were eluted from the column into the mass spectrometer using the following gradient: 4% to 35%B in 120 min, 35% to 90%B in 5 min, maintained at 90% for 5 min and then back to initial conditions.

The nanoUPLC was coupled online through a nanoESI emitter (10 μm tip; New Objective; Woburn, MA, USA) to a quadrupole orbitrap mass spectrometer (Q Exactive Plus, Thermo Scientific) using a FlexIon nanospray apparatus (Proxeon).

Data was acquired in data dependent acquisition (DDA) mode, using a Top10 method. MS1 resolution was set to 70,000 (at 400m/z), mass range of 375-1650m/z, AGC of 3e6 and maximum injection time was set to 100msec. MS2 resolution was set to 17,500, quadrupole isolation 1.7m/z, AGC of 1e5, dynamic exclusion of 40sec and maximum injection time of 150msec.

### Mass spectrometry data analysis

Raw data were analyzed in MaxQuant software (version 1.6.0.16) with the default parameters for the analysis of the proteasomal peptides, except for the following: unspecific enzyme, LFQ minimum ratio count of 1, minimum peptide length for unspecific search of 6, maximum peptide length for unspecific search of 40, and match between runs enabled. A stringent false discovery rate (FDR) of 1% was applied for peptide identification. For the analysis of tryptic digests, the default parameters were set, apart from a minimum peptide length of 6. Masses were searched against the human proteome database from UniprotKB (last update April 2020).

### Proteasome activity assay

Cells were treated either with NCT-504 (20 µM) or DMSO for 12 hours, were harvested and homogenized using a dounce homogenizer in homogenization buffer (HEPES pH 7.5: 20 mM, MgCl_2_: 1.5 mM, DTT: 0.5 mM, KCl: 10 mM). Homogenates were loaded onto a Nunc™ microwell™ 96-well flat bottom microplate (ThermoFisher, 137101) and exposed to suc-LLVY-AMC substrate (Boston Biochem, S-280) with the addition of energy mix (creatine phosphate: 375 mM, ATP: 50 mM, MgCl_2_: 50 mM). AMC fluorescence were then read by plate reader every 30 seconds over 180 minutes.

### RNA-seq

Primary mHTT MEF were treated either with NCT-504 (20 µM) or DMSO for 12 hours, then harvested. RNA was then extracted using RNeasy® plus micro kit (Qiagen, 74034), frozen at -80°C and shipped to BGI, Shenzhen, China for RNA-seq analysis. Agilent 2100 Bio analyzer (Agilent RNA 6000 Nano Kit) was used for total RNA sample QC: RNA concentration, RIN value, 28S/18S and the fragment length distribution were assessed. Differentially expressed genes were identified with DEseq2 [91]. Expression changes of over 2-fold with a P-value <0.05 were deemed significant.

### Transmission electron microscopy

Primary MEF cells were treated either with NCT-504 (20 µM) or DMSO for 12 hours and fixed with 2% glutaraldehyde (Electron microscopy sciences, 16300), 0.2 M HEPES (Gibco, 15630-080) at pH 7.4 for 30 minutes at room temperature. Fixed cells were then scraped and centrifuged at 17,000 X g for 10 minutes and left at room temperature for an additional 2 hours for fixation. Fixative buffer was then removed and fresh 0.2 M HEPES was added. Cells were stored at 4°C then processed for plastic embedding and thin sectioning as described previously [92]. The samples were imaged using a JEM-1400Plus transmission electron microscope (Jeol, Tokyo, Japan). Quantification of cytoplasmic area was performed by point counting using images taken at 3000X primary magnification, as described [93].

### EGFR internalization and degradation assay

HEK293T cells were plated onto 35 mm dishes. The following day, cells were treated with NCT-504 (20 µM) or DMSO for 12 hours. Cells were serum-starved for 1 h in the presence of 1 µg/ml cycloheximide (Sigma, C7698) and either NCT-504 or DMSO, followed by incubation with 100 ng/ml non-labeled EGF (PreProtech, AF-100-15) for 20 min at 4 °C in serum-free medium containing cycloheximide and NCT-504 or DMSO. After washing in ice-cold PBS, time point 0 was collected by cell scraping. For the 30 minute time point, cells were incubated in serum-free medium containing cycloheximide at 37 °C for 30 minutes followed by cell scraping. Cell extracts were prepared and analyzed by Western blotting.

### qPCR

Primary HDQ200 MEF cells were grown on 60mm cell culture plates were treated either with 20 µM NCT-504 or DMSO for 12 hours and then harvested. RNA was extracted using RNeasy® Plus micro kit (Qiagene, 74034) and reverse-transcribed to cDNA using HighCapacity cDNA Reverse Transcription Kit (Invitrogen, 4368814). Samples were plated on MicroAmp® optical reaction plate (Invitrogen, 4309849) with Power SYBR Green PCR Master Mix (Invitrogen, 4367659) using primers for mouse HTT and GAPDH as control. Samples were run on Quant studio 5 real time PCR machine (Applied biosystems) for ΔΔCT calculation.

Primers used:

**Table:** 

Gene	Forward primer	Reverse primer
Mouse GAPDH	GTCGGTGTGAACGGATTTG	GAACATGTAGACCATGTAGTTG
Mouse HTT	CCTCTCCTGCTTCCTTGTTAG	GTGTCTGTAGAAGTCTGTGGC
Mouse P62	GAGGCACCCCGAAACATGG	ACTTATAGCGAGTTCCCACCA
Mouse Beclin 1	TTTTCTGGACTGTGTGCAGC	GCTTTTGTCCACTGCTCCTC
Mouse GABARAP	GCCTACAGTGATGAAAGCGTCTA	GAGCCTGAAGGAGGAACTGG
Mouse UVRAG	ACATCGCTGCTCGGAACATT	CTCCACGTCGGATTCAAGGAA
Mouse GABARAPL1	GGACCACCCCTTCGAGTATC	CCTCTTATCCAGATCAGGGACC
Mouse LC3B	CCCACCAAGATCCCAGTGAT	CCAGGAACTTGGTCTTGTCCA

### Statistical analysis

All statistical analyses of collected data were carried out using GraphPad Prism software version 9.3.1. P-values are shown in asterisks where *: P-value<0.05, **: P-value<0.005, ***: P-value<0.0005, ****: Pvalue<0.00005.

## Supplementary Material

Supplemental Material
