# Supplementary figures and images for: Upregulation of the ESCRT pathway and multivesicular bodies accelerates degradation of proteins associated with neurodegeneration
Source: Autophagy Rep. 2023 Jan 22;2(1):2166722. doi: 10.1080/27694127.2023.2166722 (PMC10101321; doi:10.1080/27694127.2023.2166722)

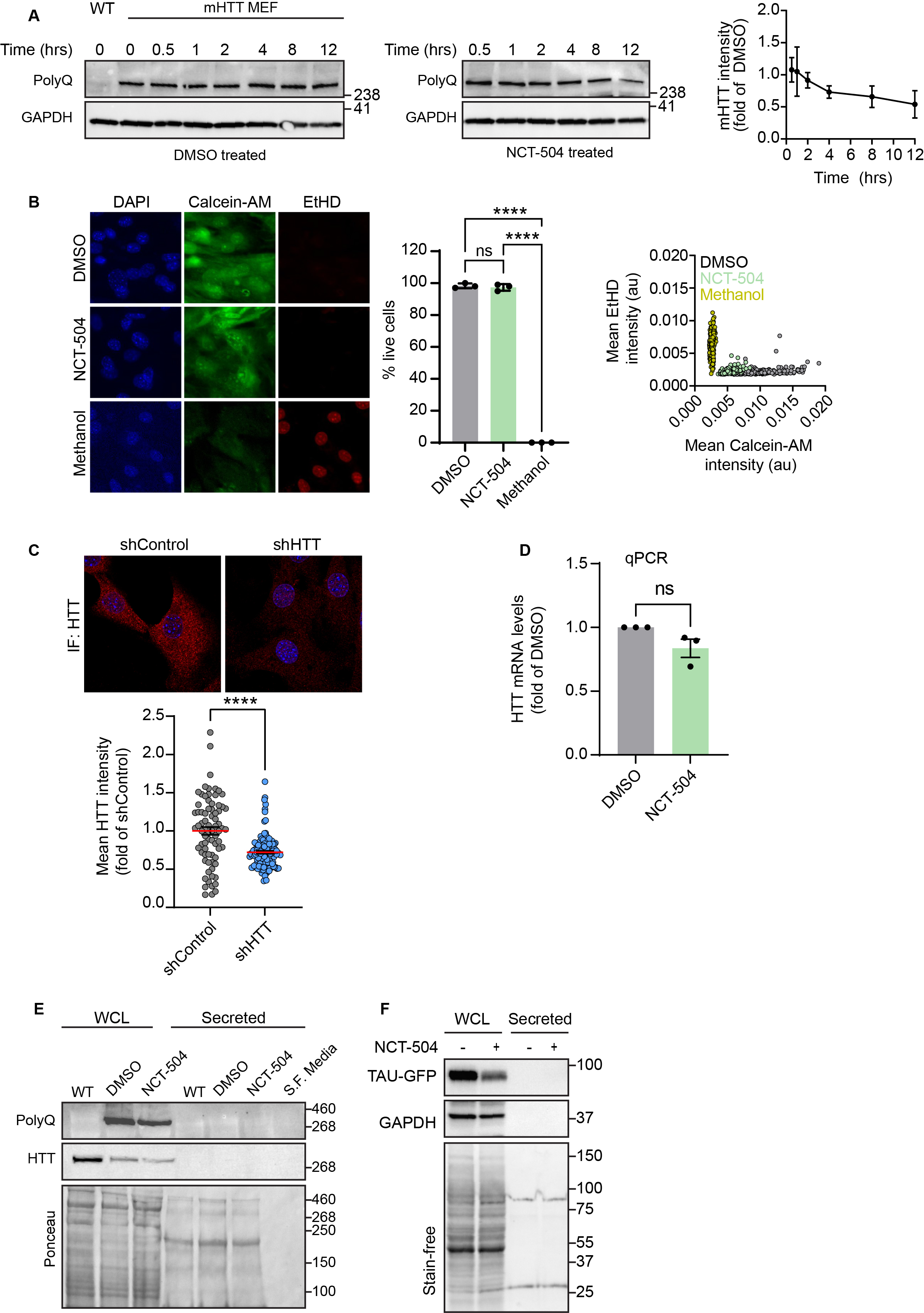

Supplement: Supplemental Material [file KAUO_A_2166722_SM3545.zip › Figure_S1 (1).jpg]

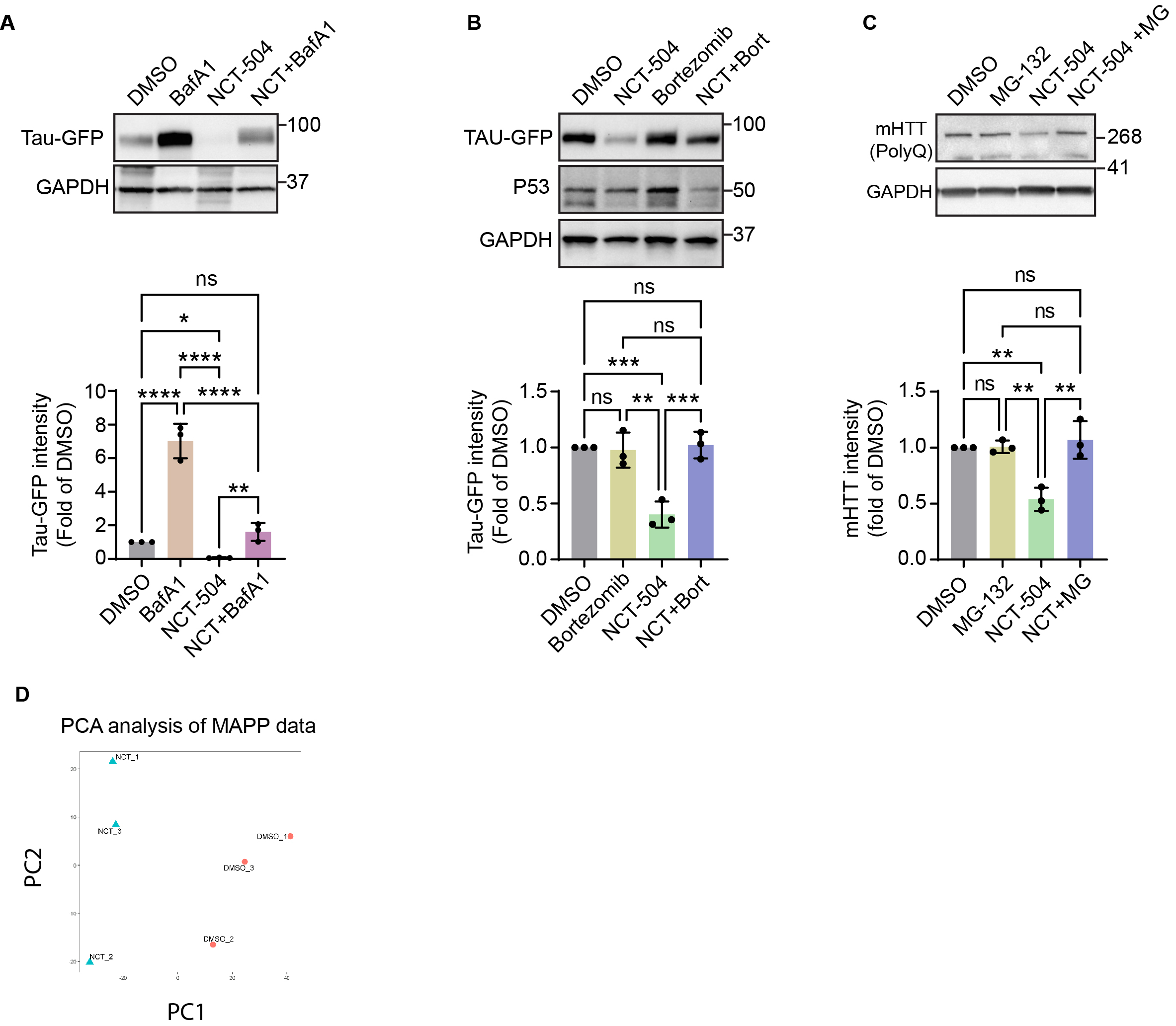

Supplement: Supplemental Material [file KAUO_A_2166722_SM3545.zip › Figure_S2 (1).jpg]

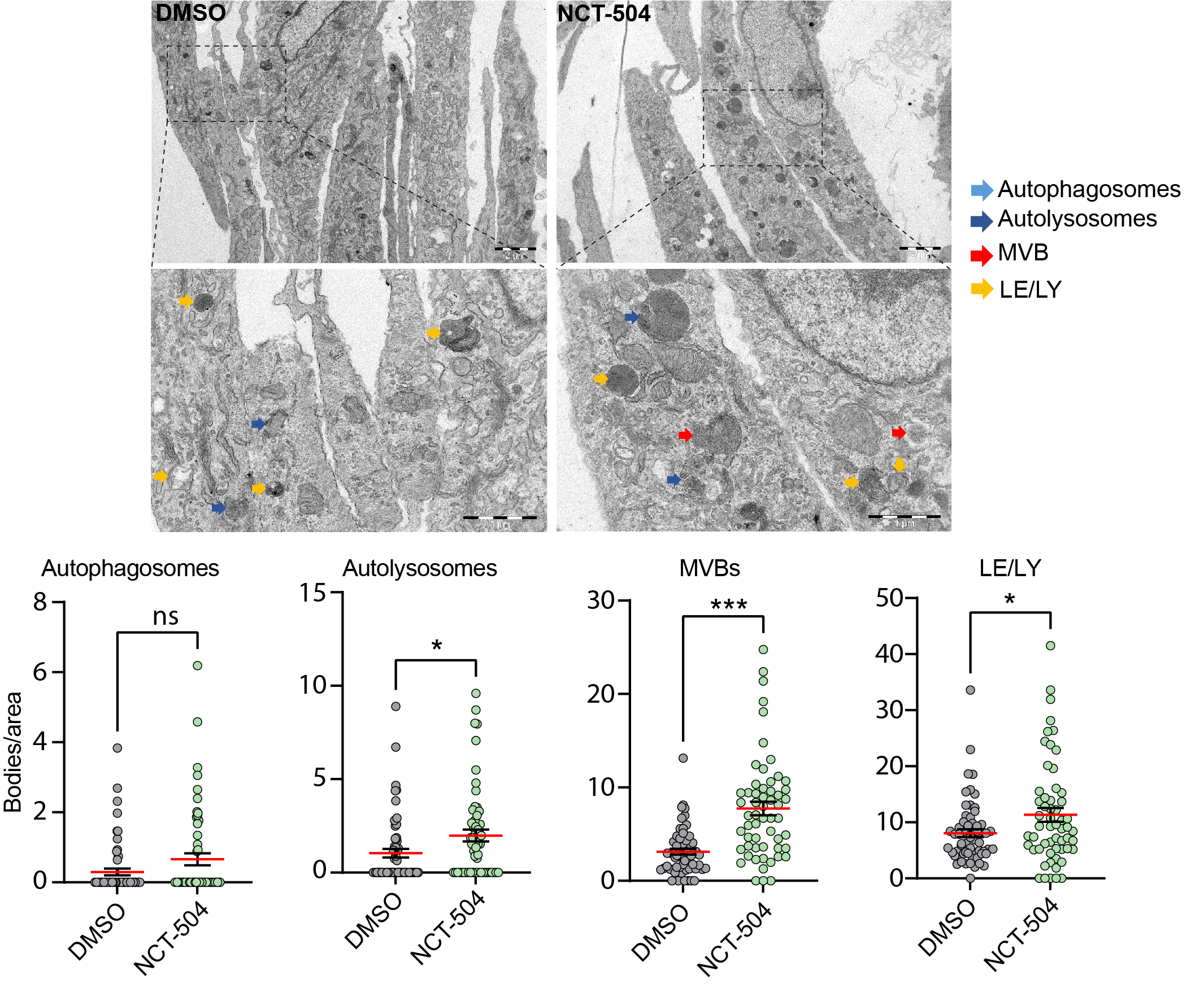

Supplement: Supplemental Material [file KAUO_A_2166722_SM3545.zip › Figure_S3 (1).jpg]

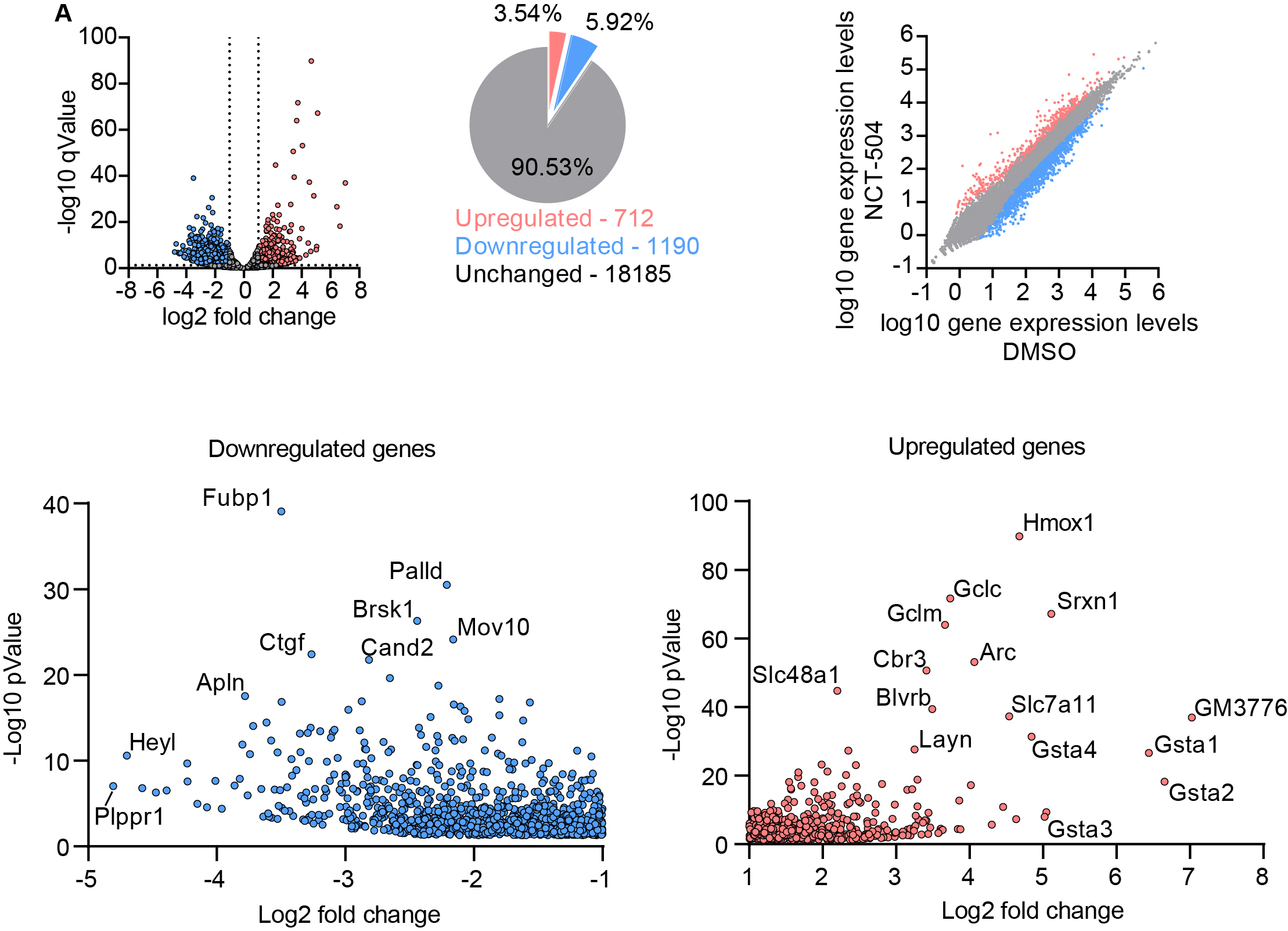

Supplement: Supplemental Material [file KAUO_A_2166722_SM3545.zip › Figure_S4.jpg]

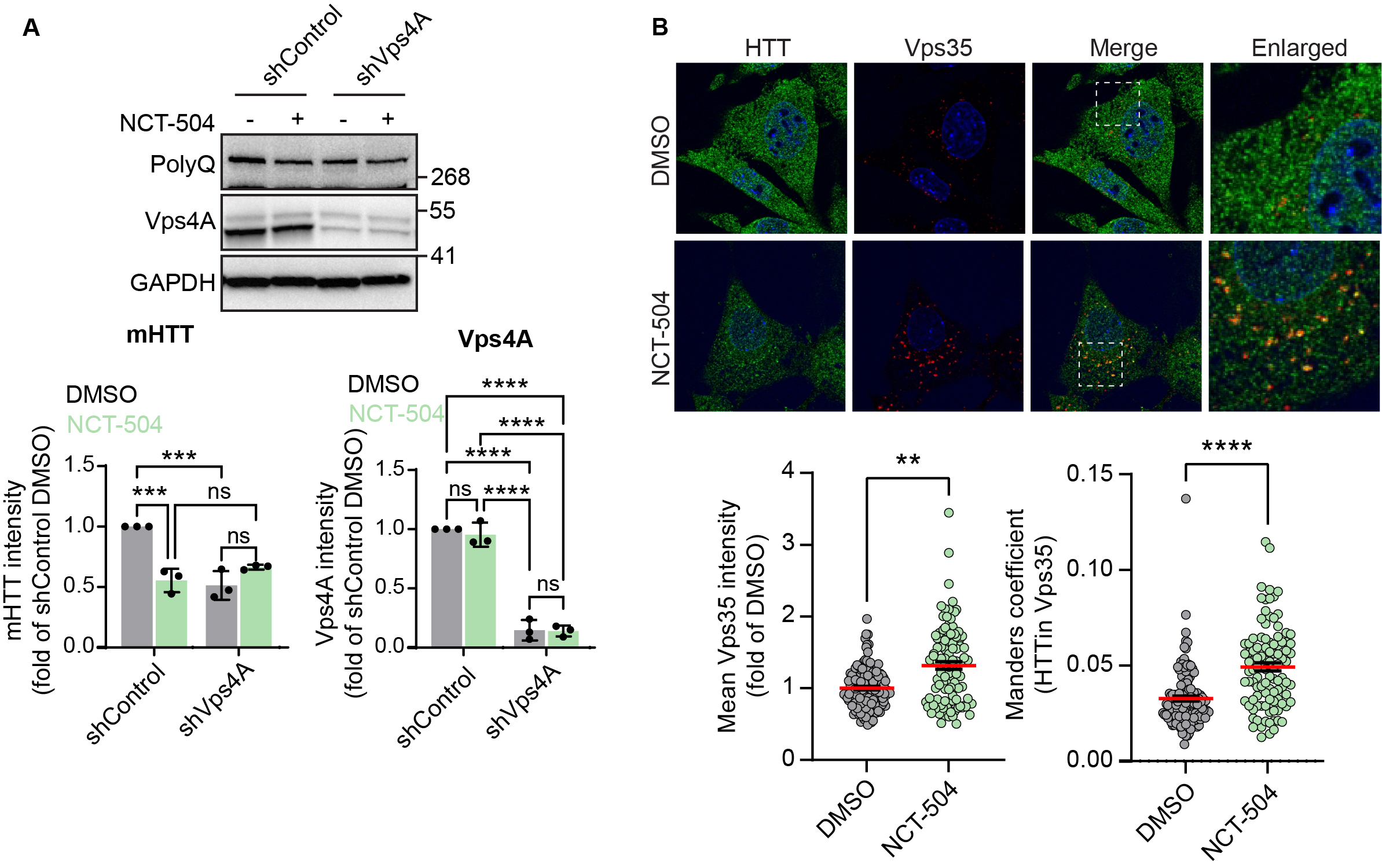

Supplement: Supplemental Material [file KAUO_A_2166722_SM3545.zip › Figure_S5.jpg]

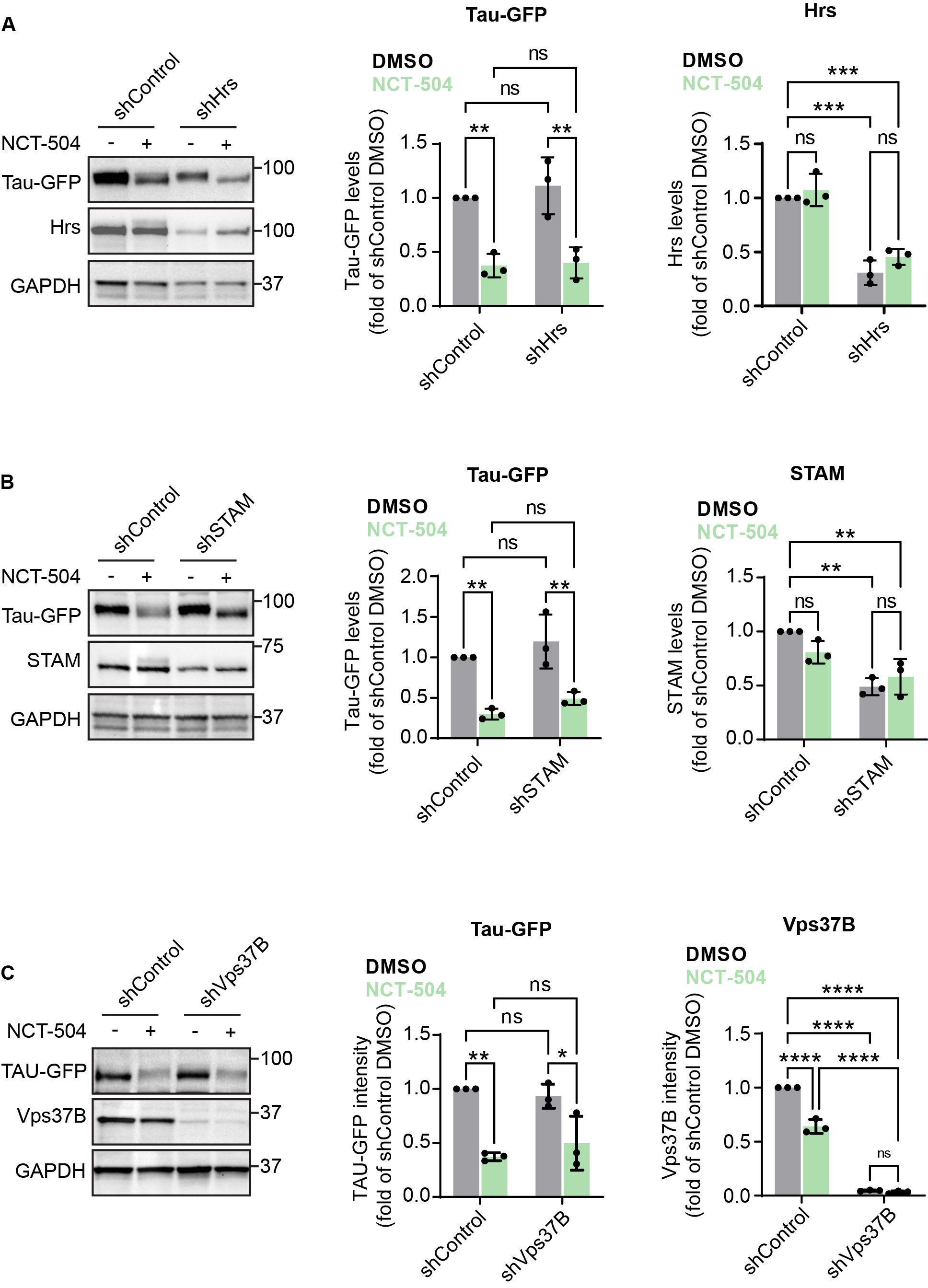

Supplement: Supplemental Material [file KAUO_A_2166722_SM3545.zip › Figure_S6.jpg]

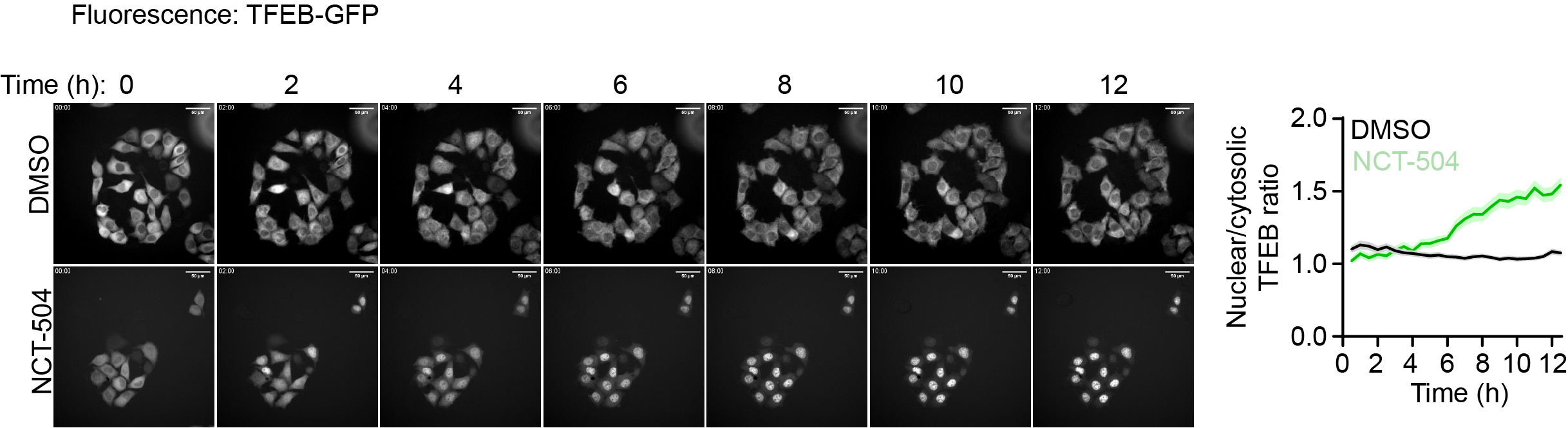

Supplement: Supplemental Material [file KAUO_A_2166722_SM3545.zip › Figure_S7.jpg]
